# Single-cell profiling reveals the trajectory of FOLR2-expressing tumor-associated macrophages to regulatory T cells in the progression of lung adenocarcinoma

**DOI:** 10.1038/s41419-023-06021-6

**Published:** 2023-08-02

**Authors:** Chan Xiang, Min Zhang, Zhanxian Shang, Shengnan Chen, Jikai Zhao, Bowen Ding, Dong Jiang, Qian Zhu, Haohua Teng, Lei Zhu, Jinchen Shao, Ruiying Zhao, Min Ye, Yang Yu, Yuchen Han

**Affiliations:** 1 Department of Pathology, Shanghai Chest Hospital, Shanghai Jiao Tong University School of Medicine, Shanghai, 200030 China; 2 Novogene Co., Ltd., Beijing, 100015 China

**Keywords:** Non-small-cell lung cancer, Cancer microenvironment

## Abstract

An immunosuppressive microenvironment enriched with regulatory CD4^+^ T lymphocytes (Tregs) facilitates the progression of lung adenocarcinoma (LUAD). This study aims to investigate the cellular mechanism underlying the formation of the immunosuppressive microenvironment in LUAD. LUAD samples (*n* = 12) and normal lung samples (*n* = 3) were obtained from patients with different pathological stages of LUAD. Single-cell RNA sequencing was performed to classify cellular components and analyze the transcriptomes, including transcription factors/targets and chemokine ligands/receptors, followed by bioinformatics study such as pseudotime analysis. Myeloid cells and T cells were the most abundant cell types in tumors and normal lung tissues, while tumor-associated macrophage-folate receptor 2 (TAM-FOLR2) and CD4^+^ nuclear receptor subfamily 4 group A member 3 (NR4A3) exhibited sharp increases in invasive adenocarcinoma (IA). The enrichment of TAM-FOLR2 in IA might result from alveolar resident macrophage-resistin (ARM-RETN) transformation and recruitment of dendritic cells (DCs) and other TAMs, as evidenced by temporal trajectories and differential expression profiles of chemokine ligands/receptors versus those in the early stages of tumors. High expression of CCL17/19/22 was observed in IA as well as in DCs, along with the strong interaction of TAM-FOLR2 with DCs. The results of pseudotime analysis suggested that CD4^+^NR4A3 might potentially convert to CD4^+^FOXP3, further supported by the high expression of NR4A3 target genes in CD4^+^FOXP3 cells. This study provides a single-cell transcriptome atlas from preinvasive to invasive LUAD and reveals a potential ARM-RETN/TAM-FOLR2/DCs/CD4^+^NR4A3/CD4^+^FOXP3 trajectory in shaping the immune suppressive microenvironment along the pathogenesis of LUAD.

## Introduction

Lung cancer is the leading cause of cancer-related deaths worldwide, with lung adenocarcinoma (LUAD) representing the most common subtype [1]. LUAD is thought to develop stepwise from atypical adenomatous hyperplasia (AAH) to adenocarcinoma in situ (AIS), to minimally invasive adenocarcinoma (MIA), and eventually to overt invasive adenocarcinoma (IA) [2]. Although several studies support the linear model for LUAD progression from AAH to AIS, MIA, and IA based on genomic alterations [2–4], the molecular landscape depicting early lung adenomatous carcinogenesis remains largely unknown.

The tumor microenvironment (TME) is a multicellular community comprising malignant epithelial cells, infiltrating immune cells, stromal cells, and other cell types that interact and collectively determine disease progression and treatment response [5]. The immune cells in the TME include macrophages, mast cells, natural killer (NK) cells, dendritic cells (DCs), as well as T and B lymphocytes. Different immune cell subsets are recruited into the TME via interactions between chemokines (CCL, CXCL, CX3CL, and XCL) and their receptors (CCR, CXCR, CX3CR, and XCR) [6, 7]. In the TME, chemokines are secreted by tumor cells and other cell types, such as immune cells and stromal cells, playing a critical role in shaping the immune cell composition and thus affecting tumor progression [8].

The immune system can create immunosuppressive conditions within the TME that allow tumor growth [9]. Results of previous studies have also shown the upregulation of some immunosuppressive markers during the progression from preneoplasia to LUAD [10, 11]. Regulatory CD4^+^ T lymphocytes (Tregs) and tumor-associated macrophages (TAMs) are prototypical immunosuppressive cell types in the TME, suppressing the function and proliferation of effector CD4^+^ and CD8^+^ T cells as well as NK cells by secreting or enhancing the secretion of immunosuppressive molecules such as interleukin-10 (IL-10) and transforming growth factor-β (TGF-β) [12–14]. Chemokine ligand secretion and chemokine receptor expression are often altered in the TME, leading to the recruitment of tumor-suppressive immune cells as well as tumorigenic immune cells, including Tregs and TAMs. Intratumoral Treg infiltration has frequently been observed in lung cancer and is associated with poor prognosis in patients [15–17]. However, the origin and trajectory of tumor-resident Tregs in LUAD remain largely unknown.

Single-cell RNA sequencing (scRNA-seq) has been successfully applied in tracking the trajectory of distinct cell lineages, investigating differentiation processes of cell populations, and uncovering new cell subsets [18, 19]. In this study, to reveal the underlying mechanism of LUAD progression, we used the scRNA-seq method to track the trajectory of immunosuppressive cells across different pathological stages of LUAD. Our results provide new information about the formation of immunosuppressive TME in LUAD progression.

## Materials and methods

### Patients and sample collection

A total of 12 LUAD tissue samples and 3 normal lung tissue samples were obtained from 12 patients who underwent pulmonary resection for LUAD at Shanghai Chest Hospital from January 2020 to August 2020. Patients were diagnosed with AIS, MIA, and IA by intraoperative frozen sections and confirmed by formalin-fixed paraffin-embedded (FFPE) blocks according to the 2015 WHO classification of lung tumors [20]. Residual surgical specimens after pathological diagnosis were used for single-cell analysis. Residual FFPE blocks containing representative tumors were further included for the TME classification by immunochemistry staining for programmed cell death 1 ligand 1 (PDL1) and CD8. The clinical characteristics of the 12 patients were summarized in Table S1. Additional 15 archived and surgically resected tumor specimens were collected from patients with 5 AIS, 5 MIA, and 5 IA as an independent cohort for the IHC validation of specific markers. None of these patients received preoperative chemotherapy or radiotherapy, or immunotherapy. This study was approved by the Ethics Committee of Shanghai Chest Hospital and conducted in accordance with the Declaration of Helsinki of 1964 and its later amendments. Written informed consent was obtained from all patients.

### scRNA library construction and sequencing

The tumor tissue and normal lung tissue were sampled after the surgical resection and transported in DMEM/F12 medium (Gibco) on ice to the research facility within 1 h. Single-cell suspensions (1 × 10^6^/mL) were submitted to 10x genomics Chromium Controller to generate single-cell gel beads in the emulsion. RNA from the barcoded cells was subsequently reverse-transcribed, and sequencing libraries were constructed with reagents from a Chromium Single Cell 3’ v2 reagent kit (10x Genomics) according to the manufacturer’s instructions. Sequencing was performed with Illumina NovaSeq 6000 platform according to the manufacturer’s instructions.

### Single-cell RNA sequencing analysis and identification of marker genes

Raw data quality control, reference mapping, barcode processing, and unique molecular identifier (UMI) counting were performed using the CellRanger v5 (10x Genomics). Reads were aligned to the GRCh38 human reference genome using STAR. Seurat package (v4.0.0) [21] was applied to perform barcode filtering and cell clustering analysis. Barcodes were removed if they had more than 20,000 UMIs or fewer than 200 expressed genes or >5% UMIs that were derived from the mitochondrial genome. Doublets were excluded using DoubletFinder software. After normalization and dimensionality reduction, Seurat canonical correlation analysis (CCA) algorithm was used to integrate 15 individual scRNA-seq datasets for batch effect correction. A summary of scRNA-seq data is shown in Table S2. The FindClusters function was adopted for cell clustering analysis with a resolution of 0.5. Uniform manifold approximation and projection (UMAP) [22] was used for the visualization of cell clusters.

We used the Seurat FindAllMarkers function to identify marker genes (differentially expressed genes) for each cluster. The marker gene lists were filtered with the following criteria: a gene should express in >10% of the cells in a cluster, the log_2_-fold-change (log_2_FC) values of the average expression between two groups (a cell cluster vs. other cells) >0.5, and adjusted *p*-value (Bonferroni correction) <0.01.

### Subcluster analysis and cell type definition

We defined cell types by integrating the enrichment of canonical marker genes, top-ranked differentially expressed genes in each cell cluster, and the global cluster distribution. Cell clusters were labeled using canonical marker genes as follows: epithelial (EPCAM and KRT8), endothelial (CDH5 and VWF), stromal (COL1A1 and ACTA2), immune (PTPRC), T cells (CD3D/E, CD8A/B, and CD4), B cells (MS4A1), plasma (JCHAIN), myeloid cells (CD68 and MSR1), and NK cells (NKG7 and KLRF1).

Epithelial, myeloid, and T cells were turned into second-round clustering to identify sub-clusters for each cell type using Seurat. In epithelial cells, we identified four major cell types, including AT1 (AGER and CLIC5), AT2 (SFTPB and MUC1), ciliated (TPPP3 and C20orf85), and club cells (SCGB1A1). In myeloid cells, we defined six major cell types, including macrophages (MRC1, C1QA, and APOE), CD14^+^ monocytes (CD14 and VCAN), CD16^+^ monocytes (FCGR3A), neutrophil (FCGR3B), mast (TPSAB1), and DC cells (CD1C). Macrophages were further divided into two major types, including alveolar resident macrophages (ARM) and tumor-associated macrophages (TAM), based on sample origins and ARM markers (FABP4, MCEMP1, and MARCO). According to specifically expressed genes, ARMs were labeled as RETN^+^ ARMs, TAMs were annotated as FOLR2^+^ and MKI67^+^ TAMs, and two minor undefined clusters (c12 and c18). In CD8^+^ T cells, we identified five cell types, including GZMK^+^ (Teff), CXCL13^+^, ZNF683^+^, KLRD1^+^, and KLRD1^+^ CCL4L2^+^ cells. In CD4^+^ T cells, six cell types were annotated, including CCR7^+^, CXCR6^+^, FOXP3^+^ (Treg), NR4A3^+^, and two other undetermined clusters (c9 and c10). Canonical marker genes used for cell type annotation are listed in Table S3.

### Definition of cell type scores and signatures

Gene set variation analysis implemented in the GSVA package (version 1.3.0) was used for gene set enrichment analysis. To evaluate the intermediate state between ARM and TAM, we used the gsva method to estimate the ARM and TAM signature scores for each cell, which were defined as the mean expression of gene signatures. The top 5 genes highly expressed in ARMs include RETN, CCL20, IFI27, FABP4, and LYZ were used to define its signature, while highly expressed SEPP1, SPP1, LGMN, RNASE1, and FOLR2 on TAMs were used to define the signature of TAMs.

### Gene regulatory network analysis

Single-cell regulatory network inference and clustering (SCENIC) python workflow (pySCENIC v0.10.0) [22] was used to investigate transcription factors (TFs) and gene regulatory networks on epithelial cells, T cells, and myeloid cells. Single-cell gene expression matrix was first filtered to exclude all genes detected in fewer than 10% of total cells. SCENIC integrates the RcisTarget TFs and hg38 genome TSS motifs to identify TF co-expression modules and binding motif enrichment. The top 10 ranked TFs were selected as the specific regulons for each cell type. AUCell package was used to compute a score for each TF module in each cell. Gene regulons were clustered and plotted using the pheatmap function in R.

### Pseudotime cell trajectory analysis

The Monocle2 (v2.18.0) method [23] was applied to infer cell trajectories for macrophages and CD4^+^ T cells separately. The “dispersion” genes were identified using estimateSizeFactors and estimateDispersions functions in monocle2 and were used to order cells. DDRTree dimensionality reduction method was applied to construct the trajectory that was plotted in two-dimensional space.

### Visualization of gene expression for chemokine ligands/receptors

Single-cell gene expression data are obtained from the Seurat R object. The averaged expression of CCLs/CCRs and CXCLs/CXCRs at the level of cell type, individual, or stage are extracted using the AverageExpression function. Genes with a summed expression value less than 0.001 are removed. The relative gene expression patterns are visualized using the pheatmap function with the following parameters: scale = "row".

### Cell-to-cell interaction network analysis

To investigate cell-to-cell interactions among different immune cell subtypes, cytokines/chemokines were extracted from receptor-ligand pairs in NicheNet. Potential interactions between any two cell types were inferred based on gene expression levels through 1000 permutation tests using CellPhone DB (v2.1.4) [24]. Cell–cell interactions within identical cellular lineages were excluded, and only gene pairs for receptor-ligand interactions in cell types of interest were visualized.

### Immunohistochemical staining

Immunohistochemical staining (IHC) was performed on tissue sections (3.5 µm thickness) cut from FFPE blocks containing representative tumors. IHC staining for SFTPB (RM370, 1:200, Abcam Cat# ab271345), TTF-1 (SPT24, 1:300, Leica Cat# TTF-1-L-CE), AGER (EPR21171, 1:100, Abcam Cat# ab216329), FOLR2 (OTI4G6, 1:150, Invitrogen Cat# MA5-26933), NR4A3 (H-7, 1:50, Santa Cruz Cat# sc-393902), FOXP3 (236 A/E7, 1:100, Abcam Cat# ab20034), PDL1 (22C3, 1:50, Dako Cat# M3666) and CD8 (SP16, ZSGB-BIO Cat# ZA-0508) was performed using the Ventana BenchMark ULTRA instrument (Ventana, AZ, USA) or a Leica BOND-III (Leica Biosystems, Nussloch, Germany) or an Agilent Dako Autostainer Link48 (Dako, Santa Clara, CA, USA) following the manufactures’ instructions. The stained slides were evaluated by two experienced pathologists corresponding to the H&E slides.

The number of NR4A3^+^, FOXP3^+^, or FOLR2^+^ cells was evaluated at five randomly chosen areas within the neoplastic region of each sample. The density of each area was calculated by dividing the number of positive cells by the area (mm^2^) of the viewed fields. The five values of density for each sample were recorded for the comparison between each group. The mean density was calculated by dividing the sum of the number of positive cells by the sum of the area. Additionally, Lymphocytes were defined as cells positive for CD8 regardless of staining intensity. The PDL1 expression was determined by tumor proportion score (TPS) according to the scoring guidelines for PDL1 22C3 pharmDx.

### Statistical analysis

All statistical analyses were performed in the R environment, version 4.0.4. Seurat package (version 4.0.5) was applied to perform normalization, clustering, and differential expression analysis of scRNA-seq data. All derivative figures were generated using the package ‘ggplot2’ or ‘pheatmap’. Cell ratio is calculated as the relative proportion of the number of one cell subset divided by the total number of all cells in the group. Log-rank tests and Kaplan–Meier plots were used for survival analysis. *P* values from comparison for continuous variables were calculated using the Wilcoxon rank sum test, values from multiple comparisons were obtained using the Kruskal–Wallis test, and values from comparison for categorical variables were calculated by the Chi-square test. All statistical significance testing was two-sided, and results were considered statistically significant at *p* < 0.05.

## Results

### Different pathological stages of LUAD exhibit heterogeneous cellular composition

**Fig. 1:**
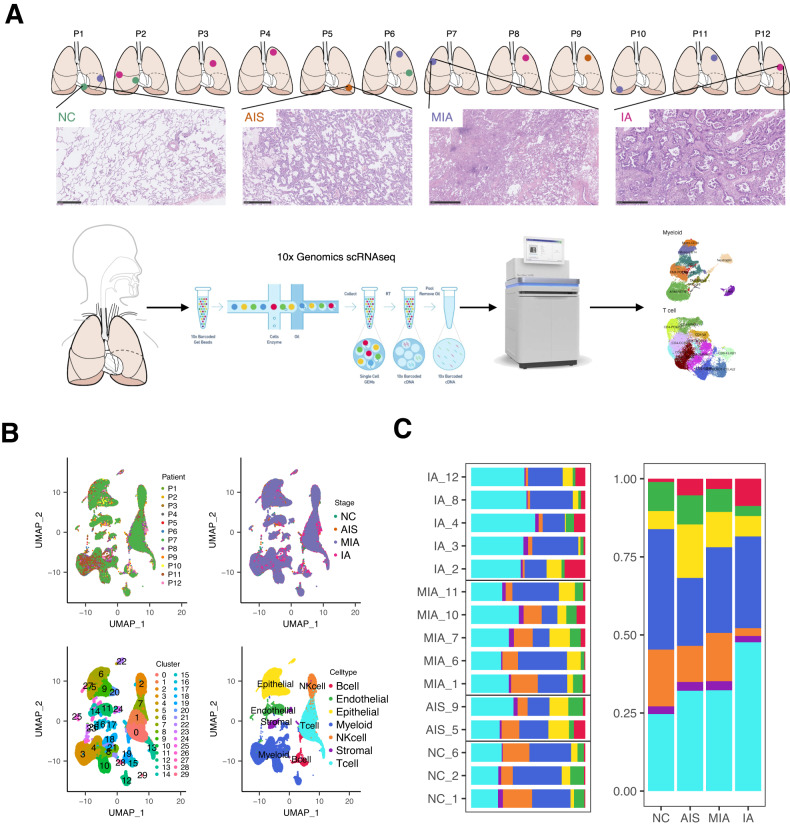
Single-cell RNA sequencing of lung adenocarcinoma (LUAD). **A** A schematic diagram of experimental workflow. A total of 12 lung tumor specimens (2 AIS, 5 MIA, and 5 IA) and 3 normal lung tissues were collected from 12 patients. Samples were then performed by 10x Genomics scRNA-seq and bioinformatic analysis. NC, normal lung tissue; AIS, adenocarcinoma in situ; MIA, minimally invasive adenocarcinoma; IA, invasive adenocarcinoma. **B** Uniform manifold approximation and projection (UMAP) of 83,662 single-cell transcriptomes color-coded by patient ID (top left), pathological stage (top right), cluster (bottom left), and cell type (bottom right). **C** Cell composition in relative cell fractions (stacked bar plot) in each patient sample (left) and each pathological stage (right). Colors correspond to the cell type annotation in B (bottom right).

To study the heterogeneity in the cellular composition of different pathological stages of LUAD, we applied scRNA-seq analysis on 12 tumor samples (2 AIS, 5 MIA, and 5 IA) and 3 normal lung samples from 12 patients with LUAD. The schematic was summarized in Fig. 1A. After quality control and filtering (Fig. S1, Table S2), the transcriptomes of 83,662 cells were analyzed. According to characteristic cell markers, we identified seven major cell types, including myeloid cells, T lymphocytes, epithelial cells, endothelial cells, NK cells, B lymphocytes, and stromal cells, with myeloid cells and T lymphocytes accounting for most of the cells (Fig. 1B, Fig. S2). The frequency of some cell types exhibited remarkable heterogeneity along the course of normal lung to preneoplasia and invasive LUAD (Fig. S2, Fig. 1C). For example, the increased relative proportion of T cells and the decline in NK cells were observed from normal lung to IA; the fraction of epithelial cells varied by histological subtype, as we observed a relatively higher frequency in tumors with lepidic pattern, but a lower frequency in tumors harboring solid/papillary pattern, and which are in line with previous observations [25]. These observations highlight transcriptomic heterogeneity in the tumor microenvironment during the malignant progression of LUAD.

Since lung epithelial cells might be involved in LUAD pathogenesis [26], we re-clustered epithelial cells and identified 4 subclusters, including alveolar type I and II (AT1 and AT2), ciliated, and club cells (Fig. S3A, Table S4). The proportion of AT2 cells was increased, whereas the proportion of AT1 cells was decreased during LUAD progression, with AT2 cells accounting for most of the cells in AIS, MIA, and IA (Fig. S3B, C). The changes were further supported at the protein level through IHC staining for AT2 markers (surfactant protein B, SFTPB; transcription termination factor 1, TTF-1) and AT1 marker (advanced glycosylation end-product specific receptor, AGER) on an independent in-house cohort with 5 AIS, 5 MIA, and 5 IA (Fig. S3D). These data suggest that LUAD mainly originates from AT2 cells, as previously reported [1]. Noticeably, survival analysis based on the Cancer Genome Atlas (TCGA) LUAD database showed neither the AT2 signature (*p* = 0.073) nor the AT1 signature (*p* = 0.530) was correlated with the prognosis of the LUAD patients (Fig. S3E). These results imply that the AT2 and AT1 signatures identified in the present study might be associated with early LUAD carcinogenesis while not being applicable to predict prognosis for invasive LUAD patients. Subsequently, we performed SCENIC to assess the differences in regulatory activities of transcription factors in epithelial cells. We found that four epithelial subclusters presented the different regulatory activity of transcription factors (Fig. S3F). While each cell type exhibited similar transcription factor expression profiles across different histological stages (Fig. S3G), suggesting that the transcription factor expression profiles might also be associated with LUAD progression. Taken together, our data indicate that different histological stages of LUAD exhibit heterogeneous cellular composition and phenotypes, which may contribute to the progression of LUAD.

### Progression of LUAD is accompanied by alterations in immune cell proportions

**Fig. 2:**
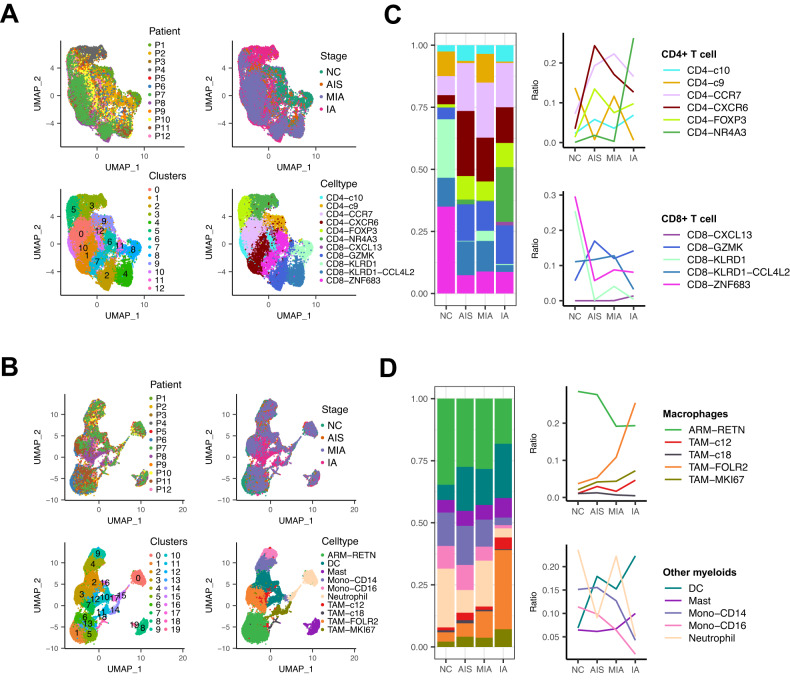
Analysis of the cellular composition of immune cells in normal and LUAD tissue samples. **A**, **B** UMAP of single-cell transcriptomes of T cells (**A**) and myeloid cells (**B**) color-coded by patient ID (top left), pathological stage (top right), cluster (bottom left), and cell type (bottom right). **C**, **D** Changes in the relative fraction of T cell (**C**) and myeloid cell (**D**) subtypes in normal lung tissues and lung tumor tissues from different LUAD stages (left, stacked bar plots; right, line plots). Colors correspond to the cell type annotation in (**A**) or (**B**) bottom right.

The large fractions of T cells and myeloid cells in LUAD samples imply their contributions to LUAD development. Thus, we re-clustered the two cell subtypes according to differentially expressed cell-state signatures (Table S5 and Table S6) and further divided them into 11 and 10 subpopulations, respectively (Fig. 2A, B). To identify the subpopulation that may be involved in LUAD progression, we quantified the fraction of each subpopulation at different histological stages. As with the previous study [10], the composition of some cell types exhibited high heterogeneity among patients (Fig. S4A and S5A), and the fractions of CD4^+^ T cells and TAMs tended to increase, whereas those of CD8^+^ T cells and ARMs tended to decrease as LUAD progressed (Fig. 2C, D). Among T cell subpopulations, the fraction of CD4^+^NR4A3 cells peaked sharply in IA, whereas other cell subclusters did not show noticeable alterations in proportion among different stages (Fig. 2C). It is noteworthy that although the enrichment of CD4^+^FOXP3 (Treg) in IA patients relative to normal lung tissues was observed in our and several earlier scRNA-seq studies [11, 27], it showed no significant increase during LUAD progression in our scRNA-seq results (Fig. 2C). This might be due to the low number of patients included in our scRNA-seq analysis. Within myeloid cells, TAM-FOLR2 cells (*p* = 0.055) and DCs (*p* = 0.041) showed an indication of enrichment in IA relative to other stages. The relative fraction of TAM-MKI67 (*p* = 0.036) was slightly elevated in IA compared to other histological stages (Fig. 2D).

The changes in some types of immune cells were further supported at the protein level by IHC staining for NR4A3, FOXP3, and FOLR2 on the in-house cohort (*n* = 15). As shown in Figs. S4B and S5B, infiltration of NR4A3 as well as FOXP3 positive cells was increased during LUAD progression (*p* < 0.05), and expression of FOLR2 was significantly higher in IA than AIS (*p* < 0.01) or MIA (*p* < 0.01), in line with the scRNA-seq data. Of note, these immune cells exhibited prominent interpatient heterogeneity as well as intratumor heterogeneity by random area counting for positive staining cells on IHC sections, as shown in Table S7. In addition, we also performed additional analyses on the single-cell dataset from Kim and colleagues’ study [28] to investigate the shifts in the composition of these immune cells from normal lung tissues to early and advanced stages of LUAD (Figs. S6 and 7). Interestingly, although no statistical difference in the cell ratio of CD4^+^NR4A3 was observed between normal lung tissues (nLung, *n* = 11) and early (tLung, *n* = 11) or late/metastatic LUAD (tL/B, *n* = 4) (*p* > 0.05), we noticed its emergence in selected tumor tissues (tLung) and brain metastases (mBrain) compared to normal lung tissues and it exhibiting high heterogeneity (Fig. S6D, E), consistent with our study. Besides, primary LUAD tissues displayed a higher proportion of CD4^+^FOXP3 than that of normal lung tissues (*p* < 0.01); late/metastatic LUAD also showed an increased median value in proportion of CD4^+^FOXP3, although no statistical difference was observed compared to that of normal lung tissues (Fig. S6E), which might be due to the low number of late/metastatic LUAD patients profiled and missed cell spectrum due to limited biopsy sampling. Moreover, the proportion of TAM-FOLR2 was enhanced in early (*p* < 0.01) as well as late/metastatic LUAD (*p* < 0.05) compared to normal lung tissues (Fig. S7D, E). The relative proportion of DCs was also observed to be increased in primary tumors compared to the normal lung tissues (*p* < 0.01; Fig. S7E). These observations, for the most part, further support our findings above.

Subsequently, we employed SCENIC to analyze the expressions of transcription factors and their target genes in each cell subtype. We found that despite the changes in the distribution of T cell subtypes during cancer development, the expression profiles of transcription factors and their target genes within the cell subtypes remain consistent (Fig. S4C, D). Similar phenomena were observed in ARM and TAM subtypes (Fig. S5C, D). These data collectively suggest that alterations in the composition of immune cells, along with the consistent expression profiles of transcription factors and target genes, are tightly associated with the progression of LUAD.

### ARM-RETN may transform to TAM-FOLR2 during the progression of LUAD

**Fig. 3:**
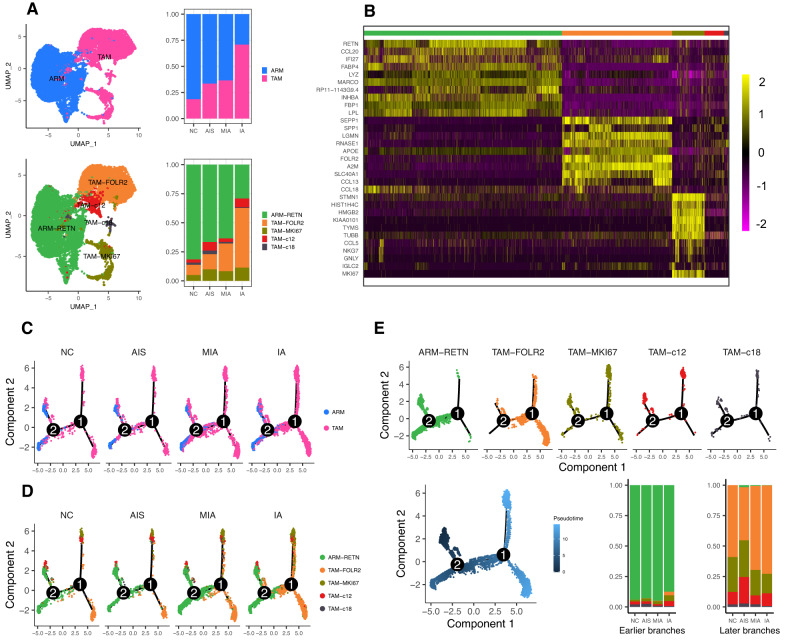
Analysis of the macrophage subtypes in normal and LUAD tissue samples. **A** Top left, UMAP classified macrophages into resident macrophages (ARMs) and tumor-associated macrophages (TAMs); top right, the relative proportions of ARMs and TAMs in normal lung and different LUAD stages; bottom left, UMAP classified macrophages into five subclusters according to signature markers**;** bottom right, the relative proportions of the subclusters in normal lung and different LUAD stages. **B** Heatmap showing the normalized expression of curated marker genes for five major macrophage subclusters. Each column represents a single cell with the corresponding cell type annotated on the top (same as in **A** bottom) as well as scaled expression level on the right. **C** Pseudotime trajectory of ARMs and TAMs from a normal cell to different LUAD stages inferred by Monocle 2 analysis. Cells on the tree are colored by cell subtypes. **D** Pseudotime trajectory of each macrophage subtype from normal tissues to different LUAD stages. Cells are color-coded by five major macrophage subtypes. **E** Trajectory analysis of five major macrophage subtypes color-coded by cell type (top panel) or pseudotime (bottom left) and the relative proportion of each macrophage subtype in earlier branches (bottom middle) or later branches (bottom right) across different pathological stages.

We further analyzed macrophage subclusters and gene-expression features along with neoplastic progression. Figure 3A top panel presented the two major macrophage subtypes, TAMs and ARMs. The proportion of TAMs was increased across different histological stages, along with a decrease in the fraction of ARM. Then, we re-clustered them into five subtypes: ARM-RETN, TAM-FOLR2, TAM-MKI67, TAM-c12, and TAM-c18 subtypes with differentially expressed markers (Fig. 3A bottom panel; Fig. 3B). The differential characteristics of TAM and ARM subclusters were further confirmed by principal coordinate analysis for TAM and ARM scores (Fig. S8A) using gene expression values (Fig. S8B). We observed that ARM-RETN and TAM-FOLR2 presented an opposite pattern in the expression of marker genes (Fig. S8C). Of note, we also found that the two cell subclusters exhibited a reverse trend in proportions with a decrease in ARM-RETN and an increase in TAM-FOLR2 along with LUAD progression (Fig. 3A bottom panel). The shifts were shown to be similar from normal lung tissues to early and late/metastatic LUAD (*p* < 0.05, Fig. S7E) by the additional analysis of the dataset from Kim and colleagues’ study [28].

Next, we performed pseudotime analysis based on Monocle2 and observed a converse pattern between ARMs and TAMs (Fig. 3C), as well as a reverse trend in the abundance of ARM-RETN and TAM-FOLR2 cells (Fig. 3D). These data collectively suggest that ARM-RETN might transform to TAM-FOLR2 during the aggressive progression of LUAD. In addition, the amounts of TAM-c12 (*p* < 0.001) and TAM-c18 (*p* < 0.001) tended to decline across AIS, MIA, and IA from later branches of pseudotime (Fig. 3E). Principal coordinate analysis showed that TAM-FOLR2 cells were well separated from ARM-RETN cells but partly overlapped with TAM-c12 and TAM-c18 subtypes (Fig. S8A), implying that TAM-c12 and TAM-c18 might be the intermediate cell types during the transformation from ARM-RETN to TAM-FOLR2.

### Chemokine recruitment of DCs and TAMs might contribute to TAM-FOLR2 elevation in IA

**Fig. 4:**
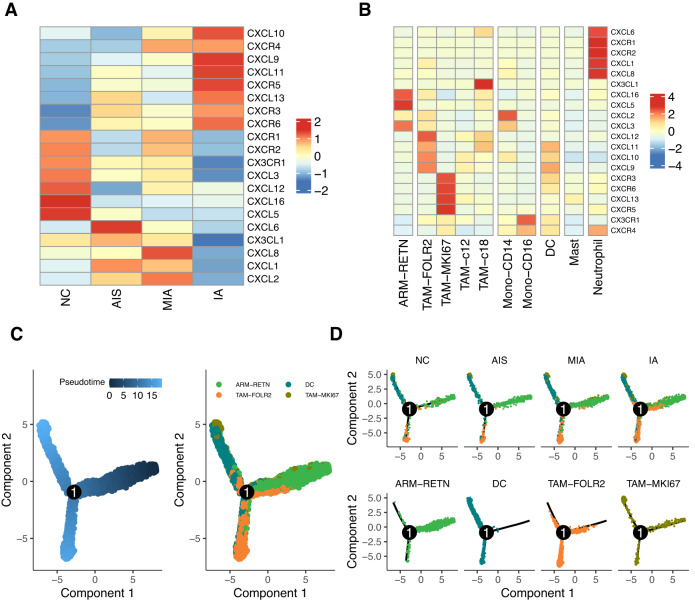
Chemokine ligand/receptor (CXCL/CXCR) expression profiles in macrophages and other myeloid cell types. **A**, **B** Heatmap of relative CXCL/CXCR expressions in macrophages of normal lung tissue samples and tissue samples of different pathological stages of LUAD (**A**) and in different myeloid cell subtypes (**B**). **C**, **D** Pseudotime trajectory of ARM-RETN, TAM-FOLR2, TAM-MKI67, and DCs color-coded by pseudotime (**C**, left) or cell type (**C**, right; **D**) along with the LUAD progression (**D**, top panel).

As shown in Fig. 2D, the decreased proportion of ARM-RETN was less than the increased proportion of TAM-FOLR2, suggesting that other cell types may contribute to the sharp increase of TAM-FOLR2 in IA. Since chemokines and chemokine receptors regulate the directed migration and positioning of immune cells [29], including macrophages, we analyzed CXCL/CXCR expressions at different histological stages of tumors. As shown in Fig. S9 and Fig. 4A, CXCL/CXCR expression profiles exhibited interpatient heterogeneity and diverse expression patterns at different pathological stages. In particular, CXCL9/10/11/13 and CXCR3/5/6 were observed with relatively high expression levels in IA (Fig. 4A). By further investigating CXCL/CXCR expressions within different myeloid cell subtypes, we observed mutually exclusive expression patterns among neutrophils, ARM-RETN, TAM-FOLR2, and TAM-MKI67 (Fig. 4B). Of note, CXCL9/10/11 presented relatively strong expression level in TAM-FOLR2 among all myeloid subtypes (Fig. 4B). These data suggest that CXCL9/10/11 and corresponding CXCRs might contribute to the enrichment of TAM-FOLR2 in IA.

To further explore the source of TAM-FOLR2 during LUAD progression, we performed pseudotime analysis. The results showed that ARM-RETN cells were mainly located in the earlier time points of the pseudotime trajectory, with DC, TAM-FOLR2, and TAM-MKI67 cells appearing later (Fig. 4C). In addition, DC, TAM-FOLR2, and TAM-MKI67 were present across AIS, MIA, and IA, with an increasing trend in abundance (Fig. 4D), suggesting that these cells might be recruited by the chemokines during LUAD progression. Thus, the enrichment of TAM-FOLR2 cells in IA possibly results from not only ARM-RETN transformation but also the recruitment of DCs and other TAMs by chemokines.

### TAM-FOLR2 might be involved in CD4^+^ T cell recruitment during LUAD progression

**Fig. 5:**
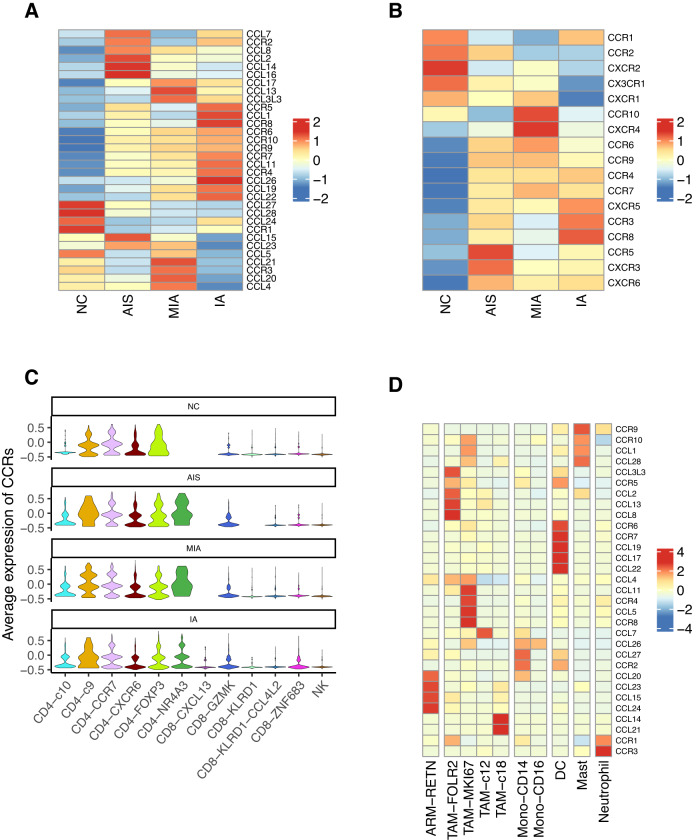
TAM-FOLR2 may be involved in the process of CD4^+^ T cell recruitment during LUAD progression. **A**, **B** Heatmap of relative expression values of chemokine ligand/receptor transcriptomes in all cells (**A**) or T cells (**B**) along the spectrum from NC to AIS, MIA, and IA stages. **C** Violin plots of average expression of chemokine receptors (CCRs) in different T cells at different pathological stages. **D** Heatmap of relative expressions of chemokine ligand/receptor transcriptomes in different myeloid subtypes.

We observed that the expressions of inflammatory cytokines, such as tumor necrosis factor (TNF), Transforming growth factor β1/2/3 (TGFB1/2/3), and interleukin 1 β (IL1B) in IA were attenuated relative to those in other stages (Fig. S10A), suggesting a varying inflammatory response or immune surveillance in the TME of IA. The elevation of TAM-FOLR2 in IA might participate in reprogramming the TME of IA. Considering the important role of T cell recruitment and infiltration in the TME of solid tumors [30], we detected CCL/CCR expressions in normal lung and tumor tissues. As shown in Fig. 5A, B, normal lung tissue and IA tissue displayed converse expression profiles of CCL/CCR and CCR/CXCRs. We noticed that the expression of CD8^+^ T cell recruitment-related CCR/CXCRs [31] (CCR5, CXCR3, and CXCR6) peaked in the AIS and attenuated thereafter (Fig. 5B). Moreover, these CCR/CXCRs were also observed with high expression in some CD8^+^ T cells (Fig. S10B). On the other hand, the relative expressions of CD4^+^ T cell recruitment-related CCR4 and CCR8 [32] were increased across AIS, MIA, and IA, consistent with the trend of TAM-FOLR2 cell fraction across different histological stages. CCR4/8 were also identified with relatively high expression in CD4^+^ T cells (Fig. S10B). Seurat analysis showed that the average CCRs expression was significantly upregulated in CD4^+^ T cells compared to that in CD8^+^ T cells throughout LUAD progression (*p* < 0.001; Fig. 5C). These data collectively suggest that the recruitment of CD4^+^ T cells but not CD8^+^ T cells might be enhanced in the progression of LUAD.

In addition, we observed that CCL5 associated with CD8^+^ T cell recruitment [33] was enriched in TAM-MKI67, whereas CCL2 related to CD4^+^ T cell recruitment [34] was robustly expressed in TAM-FOLR2, suggesting TAM-FOLR2 might be involved in the CD4^+^ T cell recruitment (Fig. 5D). Besides, we observed that there was no obviously high expression of CD4^+^ T cell recruitment-related genes (CCR4/8, CCL2) in CD8^+^ T cells (Fig. S10B) or AT2 cells (Fig. S10C). Therefore, we speculated that TAM-FOLR2 might be at least partially responsible for the recruitment of CD4^+^ T cells in IA, thereby contributing to the establishment of an immunosuppressive environment in IA.

### CD4^+^NR4A3 T cells might be recruited by TAM-FOLR2 collaborating with DCs

**Fig. 6:**
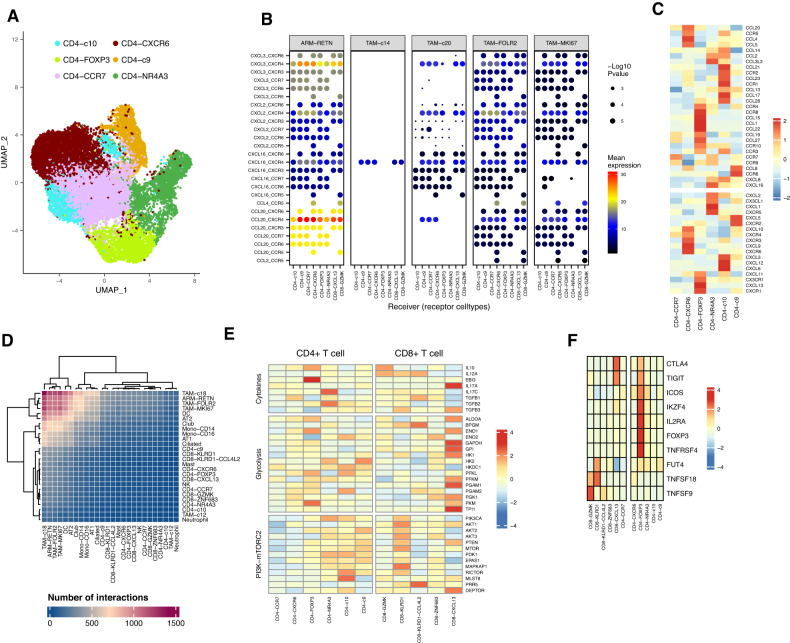
CD4^+^NR4A3 may be recruited by TAM-FOLR2 collaborating with DCs and converted to Treg. **A** UMAP of single-cell transcriptomes of CD4^+^ T cell subclusters. **B** An interaction map of selected chemokine ligands and receptors between CD4^+^ T cell subtypes and macrophage subtypes. *p-*values (two-tailed permutation test) are indicated by circle size; the scale is on the right. The means of the average level of two interacting molecules are indicated by color. **C** Heatmap of relative expression values of chemokine ligand/receptor transcriptomes in CD4^+^ T cell subtypes. **D** Heatmap depicting the interactions among different cell subtypes. **E** Heatmap of Treg targeted genes (involved in cytokines, glycolysis related, and PI3K-mTORC2 pathways) expression in CD4^+^ and CD8^+^ T cell subtypes. **F** Heatmap of NR4A3 target gene expression in CD8^+^ and CD4^+^ T cell subtypes.

To further investigate the mechanism underlying the involvement of TAM-FOLR2 in CD4^+^ T cell recruitment, we performed UMAP visualization of CD4^+^ T cells and grouped them into six subclusters, two of them were CD4^+^NR4A3 and CD4^+^FOXP3 (Treg) as we mentioned above (Fig. 6A). Since the abundance of both CD4^+^NR4A3 and TAM-FOLR2 peaked sharply in IA (Fig. 2C, D), we first sought to investigate the relationship between them. However, we did not observe a direct interaction between TAM-FOLR2 and CD4^+^NR4A3 by mapping the interactions between chemokine ligands and receptors (Fig. 6B, Fig. S11), suggesting that CD4^+^NR4A3 might be recruited by other mediators rather than directly by TAM-FOLR2. Further analysis of chemokine ligand-receptor transcriptomes of CD4^+^ T cells showed that CD4^+^FOXP3 (Treg) cells exhibited relatively high levels of CCR4/8/10 (Fig. 6C), which were also showing relatively strong expression in IA **(**Fig. 5A). As the chemokine ligand-receptor pairs of Tregs including CCL5/17/22-CCR4, CCL20-CCR6, CCL19/21-CCR7, CCL1-CCR8, and CCL27/28-CCR10 have been well-established [35], these paired chemokines were further investigated. We observed that IA also displayed relatively high expressions of CCL17/19/22 (Fig. 5A), suggesting that CD4^+^NR4A3 might be recruited in the microenvironment enriched with CCL17/19/22. As shown in Fig. 5D, CCL17/19/22 was strongly expressed by DCs. Besides, we observed a strong interaction between TAM-FOLR2 and DCs (Fig. 6D). Therefore, TAM-FOLR2 may trigger the secretion of CCL17/19/22 from DCs, which in turn recruit CD4^+^NR4A3 cells.

### CD4^+^NR4A3 might be the precursor of Treg during LUAD progression

NR4A3 has been shown to partake in the activation of Treg signatures, including FOXP3 [36]. We also noted that the CD4^+^NR4A3 and CD4^+^FOXP3 (Treg) clusters were closely related and partly overlapped (Fig. 6A). Therefore, we assumed that CD4^+^NR4A3 might be the precursor of Treg in LUAD progression. Next, we performed pseudotime analysis of CD4^+^ T cell subclusters and observed an overlap between CD4^+^NR4A3 and CD4^+^FOXP3 (Treg) clusters (Fig. S12A). Trajectory paths revealed that CD4^+^NR4A3 cells were enriched in the earlier time points and were attenuated thereafter, whereas CD4^+^FOXP3 cells were enriched in later time points, suggesting CD4^+^NR4A3 might convert to CD4^+^FOXP3 (Treg) during LUAD progression. Additionally, the high expression of Treg differentiation-pertinent TGFB1/2/3 in CD4^+^NR4A3 cells (Fig. 6E) and CD4^+^NR4A3 target genes including Eos (IKZF4), IL2RA, CTLA4, TIGIT, ICOS, and FOXP3 in CD4^+^FOXP3 cells further supported this assumption (Fig. 6F). Furthermore, we observed that glycolysis-related genes, i.e., PGK1, PKM, and GAPDH were highly expressed in CD4^+^FOXP3 cells (Fig. 6E). Increased expression of these genes also facilitates Treg differentiation in IA possibly by regulating FOXP3 expression [37, 38]. Noticeably, IL-10 and EBI3 showed relatively higher expression levels in CD4^+^FOXP3 than CD4^+^NR4A3, implying their potential roles in the conversion from CD4^+^NR4A3 to CD4^+^FOXP3 (Treg) (Fig. 6E).

## Discussion

Tumor associated-Tregs are present in multiple tissues of patients with LUAD, including tumors, metastatic lymph nodes, and the peripheral blood, playing a fundamental role in creating an immunosuppressive microenvironment in LUAD [39]. Tumor-resident Tregs may originate from peripheral Treg recruitment, local differentiation from naive T cells, the conversion of conventional T cells, or tissue Treg expansion [40]. In this study, to track the TME along with the neoplastic progression of LUAD, we applied the scRNA-seq technique to classify the cellular components of preneoplasia to invasive LUAD. We demonstrated that intratumoral Tregs in invasive LUAD may originate from CD4^+^NR4A3 cells in an environment enriched with CCL17/19/22 secreted by DCs, which might be triggered by TAM-FOLR2. This study provides new insights into the mechanism underlying the progression of LUAD from the perspective of the formation of immunosuppressive TME.

As with previous scRNA-seq studies [25, 28], we observed that the proportion of epithelial cells was lower than that of immune cells, suggesting a possibility that epithelial cell transcriptomes might be underestimated by scRNA-seq due to well-known disparities in dissociation efficiency of different cell types following tissue disaggregation [41]. Another possibility might be the tissues we used in the present study were mainly early-stage LUAD samples, showing a relatively smaller composition of epithelial cells compared to other solid tumors [42, 43]. AT2 cells are considered the cell-of-origin of LUAD [1]. Consistently, our scRNA-seq results showed that the proportion of AT2 cells accounted for most of the epithelial cells in the samples and was increased along with neoplastic progression, suggesting that LUAD mainly originates from AT2 cells. IHC staining for signature makers of AT2 cells on an in-house cohort with different pathological stages of LUAD further supports this conclusion.

Our scRNA-seq data showed that T lymphocytes and myeloid cells accounted for the majority of immune cells, in line with previous studies [25, 28]. After re-clustering T cells and myeloid cells, we observed dramatic increases in the fractions of CD4^+^NR4A3 and TAM-FOLR2 cells in IA relative to those in other stages. These cellular dynamic changes along the LUAD progression were further validated at the protein level by IHC staining on the in-house early-stage LUAD cohort and partly supported by an in-depth analysis of another published dataset with comprehensive single-cell profiling of LUAD from early to advanced stages of LUAD [28]. Loss of NR4A3 and other NR4A receptors in T cells blocks the development of Tregs, resulting in autoimmune diseases in multiple organs [44]. The NR4A proteins, including NR4A3, can regulate Treg development through the activation of FOXP3 and have therapeutic potential in immune disorders and cancer [45]. The FOLR2 gene that encodes folate receptor 2 is overexpressed in M2-polarized TAMs in lung cancer [46, 47]. It was mainly expressed in the stromal macrophages of LUAD, as supported by our IHC staining and prior study [47]. Several studies have highlighted the important roles of TAM-FOLR2 in tumor immunity [47–51]. Of note, TAM-FOLR2 might be endowed with distinct roles in cancer progression and antitumor immunity. Buggattie and colleagues presented two distinct macrophage subsets co-expressed with FOLR2 and TIM4, which may have opposing roles in tumorigenesis and tumor immunity across several cancer types [51]. These studies, as well as our current study, collectively highlight TAM-FOLR2 as an attractive therapeutic target in cancer treatment. Taken together, co-occurring increases in the abundance of CD4^+^NR4A3 and TAM-FOLR2 in IA imply the involvement and collaboration of both cell subtypes in the evolution of the immune microenvironment of IA.

Then, we sought to establish the connection between TAM-FOLR2 and CD4^+^NR4A3. Due to the lack of evidence on a direct TAM-FOLR2/CD4^+^NR4A3 interaction, we sought to identify a mediator that may recruit CD4^+^NR4A3. T cell-attracting chemokines are secreted by various cell types, including tumor cells, DCs, and macrophages [52]. By combining the scRNA-seq data regarding chemokine ligand/receptor profiles in different stages of tumors and cell subtypes, we found that DCs may attract CD4^+^NR4A3 by secreting CCL17/19/22. CCL17, CCL19, and CCL22 have been shown to mediate DC trafficking between the tumor and draining lymph nodes while promoting the activation of tumor-specific T cells [53]. CCL17 and CCL22 can also attract Tregs in certain cancers [54]. Therefore, the microenvironment enriched with CCL17/19/22 might not only recruit CD4^+^NR4A3 but also facilitate the establishment of an immunosuppressive TME. Kim et al. have classified the tumor immune microenvironment into four different types according to the expression of PDL1 and the presence of infiltrating lymphocytes (mainly based on CD8^+^ T cells) in tumor samples [55]. Hereby, cases in our scRAN-seq cohort were subjected to IHC staining for PDL1 and CD8. All of them showed infiltration of lymphocytes but lacked PDL1 expression (TPS < 1%) and were classified as type III- immune tolerance (Table S1), indicating the presence of other non-PD1–PDL1 adaptive immune resistance mechanisms in promoting immune tolerance. The immune axis observed in our study might partly contribute to immune tolerance, suggesting a value in combinatorial targeting of multiple markers in TME for immunotherapy of invasive LUAD to break the immune tolerance.

Naïve T cells can be converted into Tregs through IL-2 and TGF-β stimulation [56, 57]. Our results showed that TGFB1/2/3 expression was relatively higher in MIA compared to those in IA, suggesting that Treg differentiation may occur in MIA and promote the progression from MIA to IA. Forced expression of FOXP3 converts naïve T cells toward a Treg phenotype similar to that of naturally occurring Tregs [58]. NR4A3 induces the expression of FOXP3 and thus promotes Treg differentiation [44]. We observed high expression of TGFB1/2/3 in CD4^+^NR4A3 cells, suggesting that CD4^+^NR4A3 may potentially convert into Tregs. The high expression of CD4^+^NR4A3 target genes in CD4^+^FOXP3 cells further suggests that the intratumoral Tregs in invasive LUAD might originate partly from CD4^+^NR4A3 cells. Interestingly, we noticed a strong expression of CXCL13 in the CD4^+^FOXP3 subtype, suggesting a link between CD8^+^CXCL13 and Tregs. Despite accounting for a small percentage of Tregs, the CD8^+^FOXP3^+^ Treg subtype is indeed present in the TME of LUAD [59], meriting further investigation. Since the activation of CD4^+^ T cells requires interaction with the antigen-major histocompatibility complex class (MHC) complex [60], we also analyzed the MHC expression on different cells (Fig. S12B–D). We observed abundant expression of MHC-II on TAM-FOLR2, which was consistent with an increase of CD4^+^ T cells in IA. Given the evidence we presented, however, whether TAM-FOLR2 might contribute to CD4^+^ T cell differentiation remains to be interrogated in future studies.

This study has some limitations. First, due to the small preinvasive lesions, which hindered the acquisition of enough cells per patient for subsequent scRNA-seq, the number of patients, particularly AIS patients, included in this study was low. Second, the recruitment of CD4^+^NR4A3 by DCs and the conversion between different cell phenotypes were speculated based on biomarker expressions and pseudotemporal estimation, warranting further validation through cell-based experiments.

In summary, we provided a single-cell transcriptome atlas from preinvasive to invasive LUAD, which comprises high cellular heterogeneity in cell subclusters and their transcriptomic features at different pathological stages. In addition, we first reported the possible trajectory of FOLR2-expressing TAMs to regulatory T cells during the progression of LUAD. The results of our study may unveil the potential of ARM-RETN/TAM-FOLR2/DCs/CD4^+^NR4A3/CD4^+^FOXP3 trajectory in shaping the immune suppressive microenvironment in invasive LUAD.

### Supplementary information

Supplementary Figures

Supplementary Tables

Reproducibility checklist

## Data Availability

Raw sequencing data in this study can be accessed after an approved application to the Genome Sequence Archive for Humans (https://ngdc.cncb.ac.cn/gsa/) under the accession number HRA003685. The main data supporting the results are available within this article and its supplementary information. Other data used in this study can be obtained by request to the corresponding author. The TCGA LUAD dataset was downloaded from cBioPortal (https://www.cbioportal.org). The cited single-cell dataset of Kim and colleagues’ study [28] was downloaded from the NCBI Gene Expression Omnibus database (accession code GSE131907).

## Code availability

The codes generated during this study can be accessed via (https://github.com/zanmer/TAM_FOLR2_scRNA_LUAD_progression), DOI (10.5281/zenodo.7972572).
