# Correction: Single-cell profiling reveals the trajectory of FOLR2-expressing tumor-associated macrophages to regulatory T cells in the progression of lung adenocarcinoma

**DOI:** 10.1038/s41419-023-06401-y

**Published:** 2024-02-14

**Authors:** Chan Xiang, Min Zhang, Zhanxian Shang, Shengnan Chen, Jikai Zhao, Bowen Ding, Dong Jiang, Qian Zhu, Haohua Teng, Lei Zhu, Jinchen Shao, Ruiying Zhao, Min Ye, Yang Yu, Yuchen Han

**Affiliations:** 1 Department of Pathology, Shanghai Chest Hospital, Shanghai Jiao Tong University School of Medicine, Shanghai, 200030 China; 2 Novogene Co., Ltd., Beijing, 100015 China

**Keywords:** Non-small-cell lung cancer, Cancer microenvironment

Correction to: *Cell Death and Disease* 10.1038/s41419-023-06021-6, published online 02 August 2023

In this article the funding number from the National Natural Science Foundation of China is currently No. 820031524 (CX).

It should be read:

The funding number from the National Natural Science Foundation of China is No. 82003154 (CX).

The original article has been corrected

