# Supplementary material for: Single-cell profiling reveals the trajectory of FOLR2-expressing tumor-associated macrophages to regulatory T cells in the progression of lung adenocarcinoma
Source: Cell Death Dis. 2023 Aug 2;14(8):493. doi: 10.1038/s41419-023-06021-6 (PMC10397223; doi:10.1038/s41419-023-06021-6)
Supplement: Supplementary file 1 — Supplementary Figures [file 41419_2023_6021_MOESM1_ESM.pdf]

Figure S1

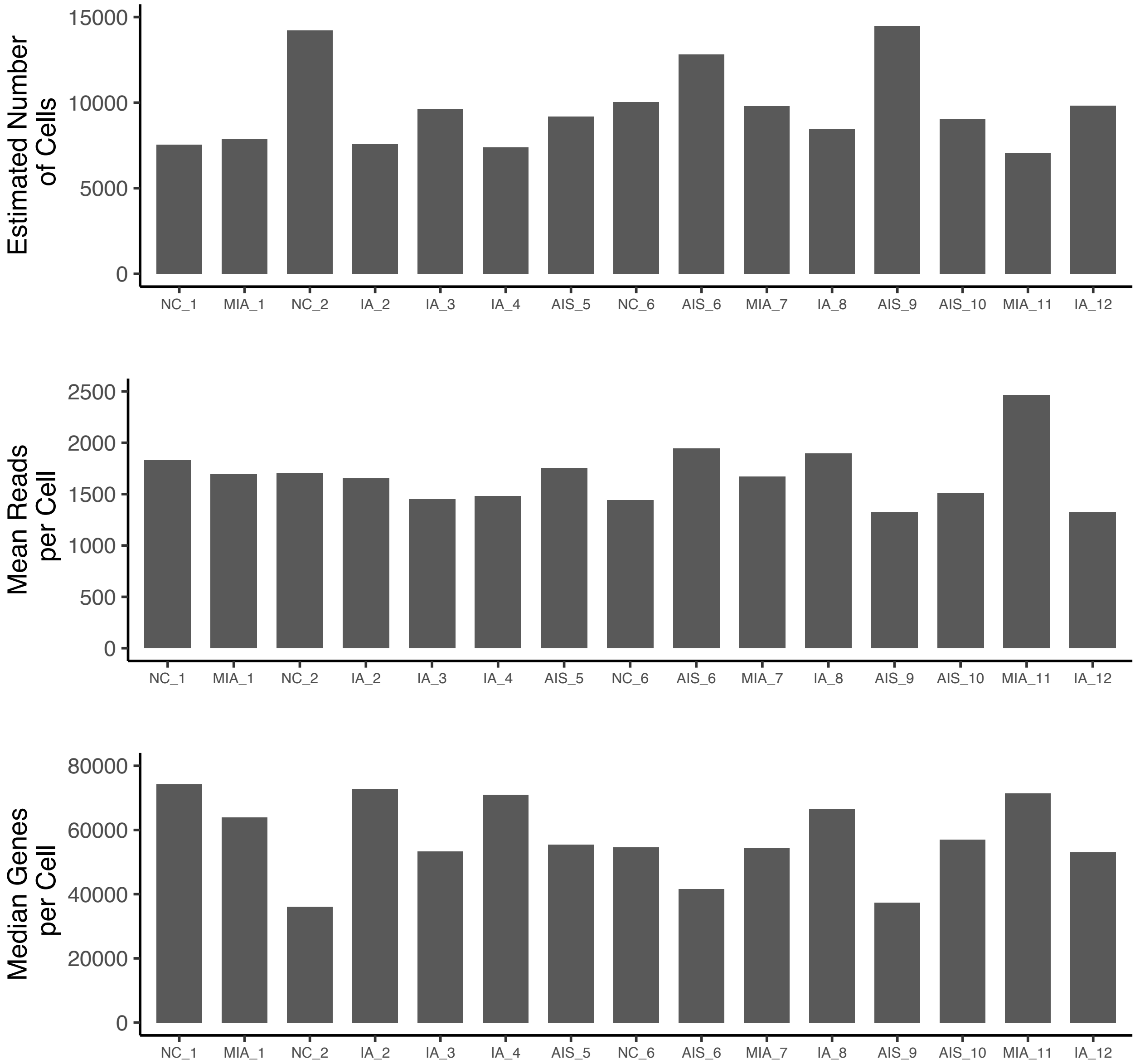

Figure S1 Quality control of the sequencing samples. The estimated number of cells passing quality control (top), mean reads per cell (middle), and the number of median genes per cell of each sample (bottom).

Figure S2

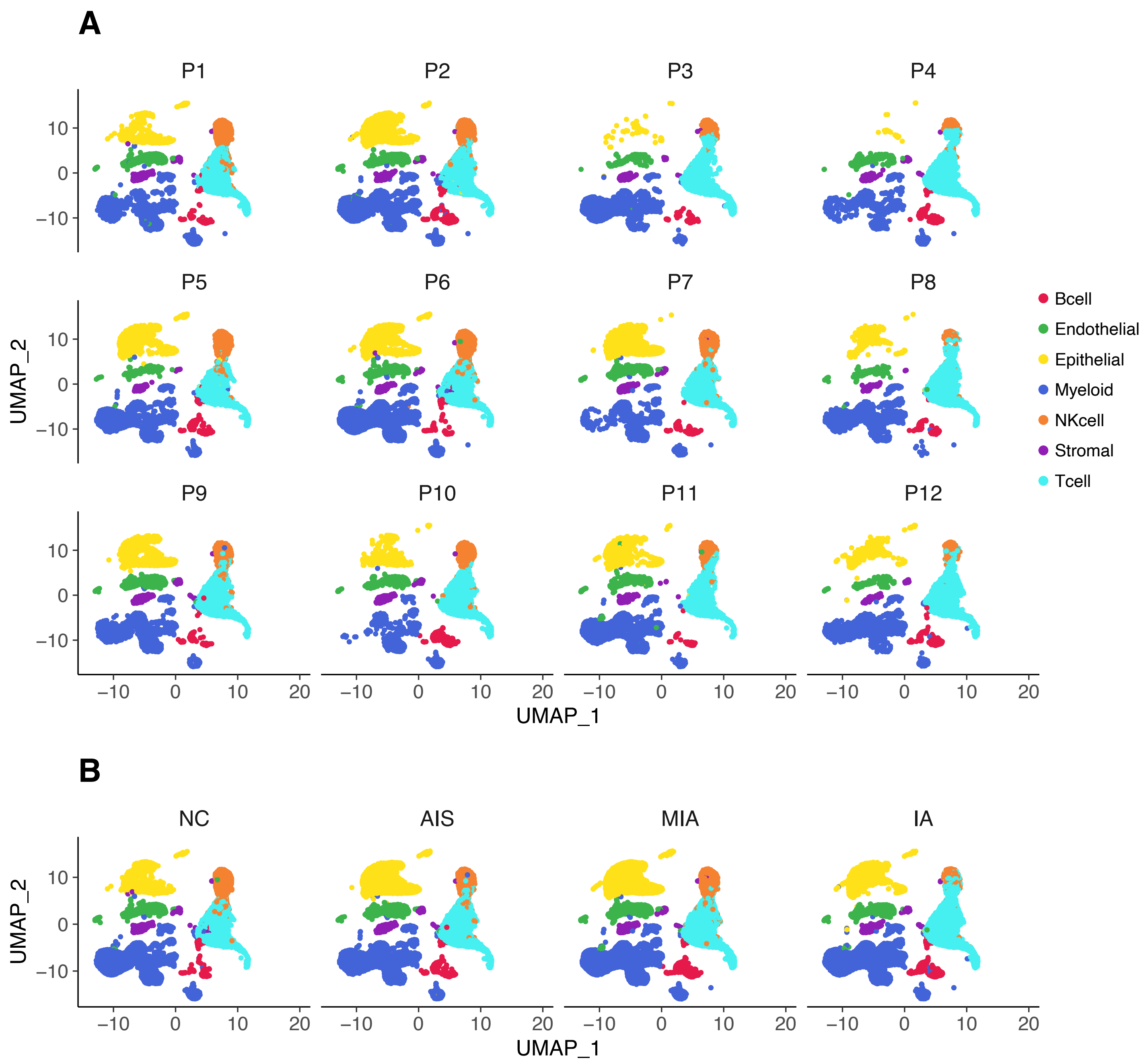

Figure S2 Origins of all cell types in 12 LUAD tissue samples. A: UMAP visualization of all 12 LUAD patients, color–coded by cell type. B: UMAP visualization of LUAD stages (AIS, MIA, IA and normal lung tissue), color–coded by cell type.

Figure S3

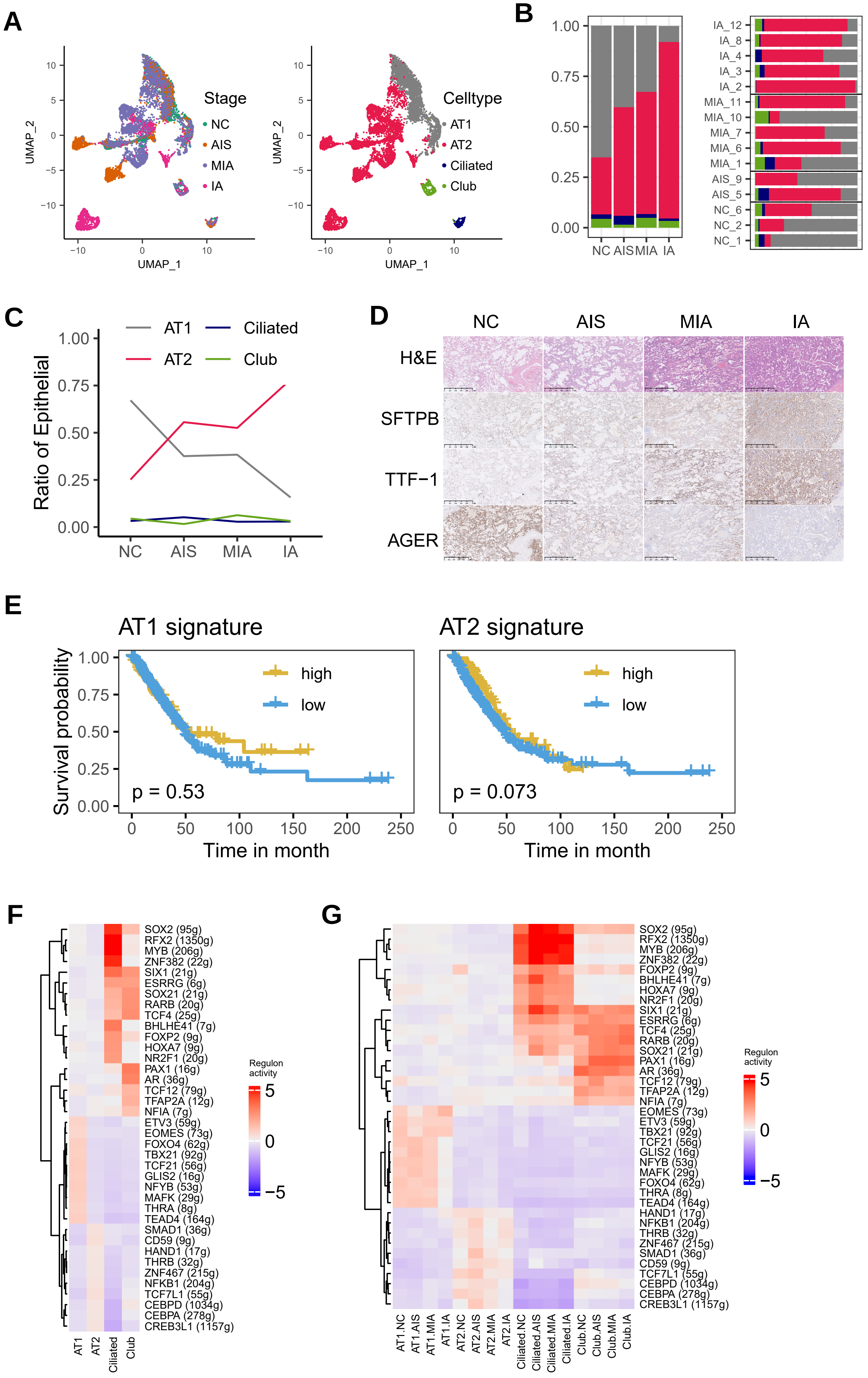
A
Stage
NC
AIS
MIA
IA
UMAP_1
UMAP_2
Celltype
AT1
AT2
Ciliated
Club
B
NC AIS MIA IA
IA_12
IA_8
IA_4
IA_3
IA_2
MIA_11
MIA_10
MIA_7
MIA_6
MIA_1
AIS_9
AIS_5
NC_6
NC_2
NC_1
C
AT1
Ciliated
AT2
Club
Ratio of Epithelial
NC AIS MIA IA
D
NC AIS MIA IA
H&E
SFTPB
TTF−1
AGER
E
AT1 signature
AT2 signature
high
low
Survival probability
p = 0.53
p = 0.073
Time in month
F
G
Regulon activity
SOX2 (95g)
RFX2 (1350g)
MYB (206g)
ZNF382 (22g)
SIX1 (21g)
ESRRG (6g)
SOX21 (21g)
RARB (20g)
TCF4 (25g)
BHLHE41 (7g)
FOXP2 (9g)
HOXA7 (9g)
NR2F1 (20g)
PAX1 (16g)
AR (36g)
TCF12 (79g)
TFAP2A (12g)
NFIA (7g)
ETV3 (59g)
EOMES (73g)
FOXO4 (62g)
TBX21 (92g)
TCF21 (56g)
GLIS2 (16g)
NFYB (53g)
MAFK (29g)
THRA (8g)
TEAD4 (164g)
SMAD1 (36g)
CD59 (9g)
HAND1 (17g)
THRB (32g)
ZNF467 (215g)
NFKB1 (204g)
TCF7L1 (55g)
CEBPD (1034g)
CEBPA (278g)
CREB3L1 (1157g)
AT1 AT2 Ciliated Club
AT1.NC AT1.AIS AT1.MIA AT1.IA AT2.NC AT2.AIS AT2.MIA AT2.IA Ciliated.NC Ciliated.AIS Ciliated.MIA Ciliated.IA Club.NC Club.AIS Club.MIA Club.IA

Figure S3 Analysis of the cellular composition of epithelial cells.

A: UMAP of all epithelial single−cell transcriptomes color−coded by pathological stage (left) and cell type (right). B and C: Bar (B) and line (C) plots of relative fractions of epithelial cell subtypes in each pathological stage and each participant. D: Representative images of H&E and IHC staining for AT2 markers (SFTPB and TTF−1) and AT1 marker (AGER) at different pathological stages from NC, to AIS, MIA, and IA. Images of NC were captured from adjacent normal tissue of AIS. Cases in the same group showed similar IHC staining results. Scale bar, 1 mm. E: Kaplan−Meier overall survival curves of TCGA LUAD patients (n=510). The high or low group was divided by the upper quantile of AT1 or AT2 signature scores (AT1 signature high n=128 and low n=382; AT2 signature high n=382 and low n=128) by integrating the 10 top−ranked significantly differentially expressed genes in each cluster of this study. P−value (p) was calculated using the two−sided log−rank test. F−G: Heatmap of gene expression regulation by transcription factors in different cell types (F) and tumor stages (G) for the epithelial cells using SCENIC (Single−cell regulatory network inference and clustering).

Figure S4

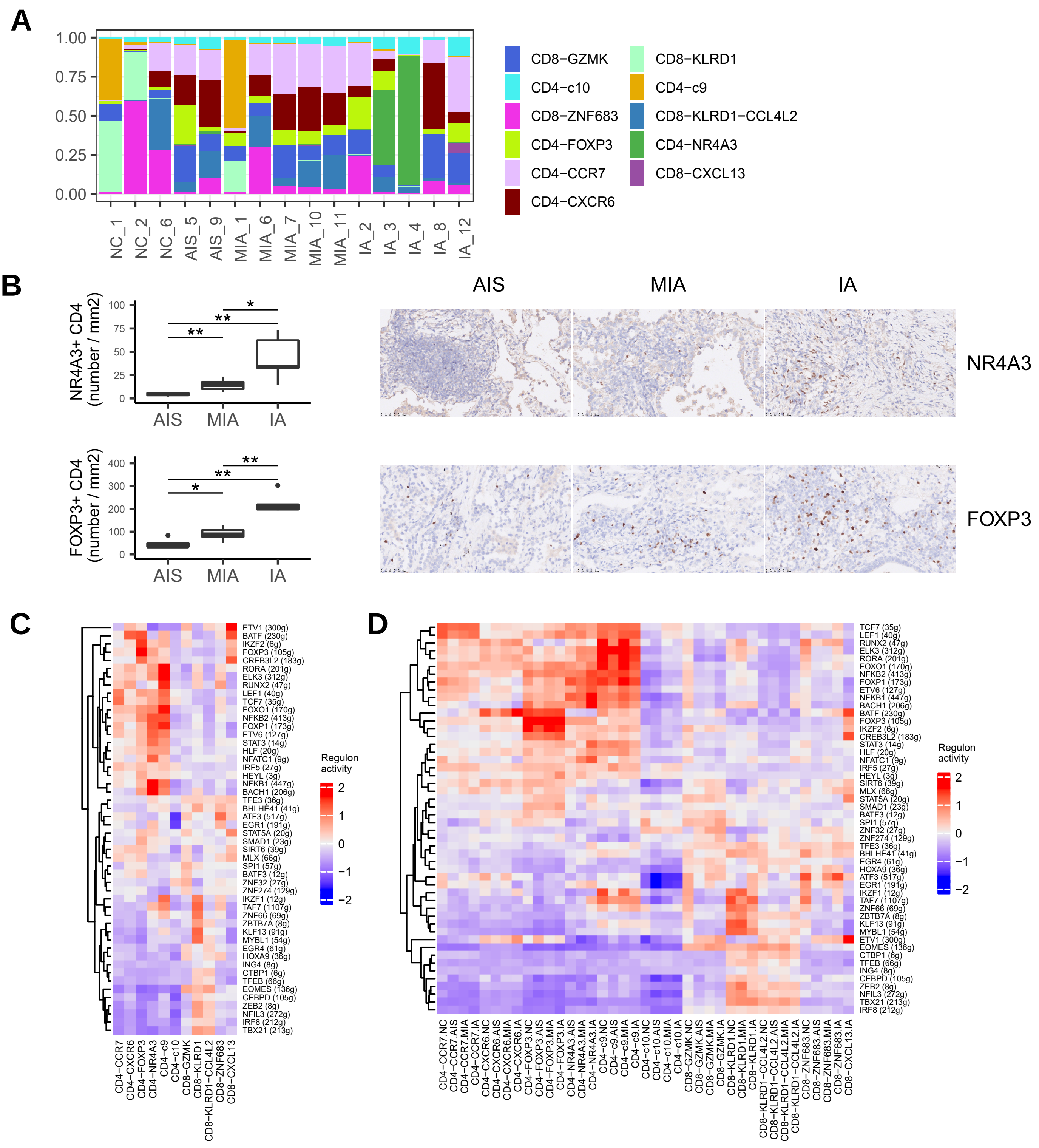

A
1.00
0.75
0.50
0.25
0.00
NC_1 NC_2 NC_6 AIS_5 AIS_9 MIA_1 MIA_6 MIA_7 MIA_10 MIA_11 IA_2 IA_3 IA_4 IA_8 IA_12
CD8−GZMK
CD4−c10
CD8−ZNF683
CD4−FOXP3
CD4−CCR7
CD4−CXCR6
CD8−KLRD1
CD4−c9
CD8−KLRD1−CCL4L2
CD4−NR4A3
CD8−CXCL13
B
NR4A3+ CD4 (number / mm2)
100
75
50
25
0
AIS MIA IA
FOXP3+ CD4 (number / mm2)
400
300
200
100
0
AIS MIA IA
AIS
MIA
IA
NR4A3
FOXP3
C
ETV1 (300g)
BATF (230g)
IKZF2 (6g)
FOXP3 (105g)
CREB3L2 (183g)
RORA (201g)
ELK3 (312g)
RUNX2 (47g)
LEF1 (40g)
TCF7 (35g)
FOXO1 (170g)
NFKB2 (413g)
FOXP1 (173g)
ETV6 (127g)
STAT3 (14g)
HLF (20g)
NFATC1 (9g)
IRF5 (27g)
HEYL (3g)
NFKB1 (447g)
BACH1 (206g)
TFE3 (36g)
BHLHE41 (41g)
ATF3 (517g)
EGR1 (191g)
STAT5A (20g)
SMAD1 (23g)
SIRT6 (39g)
MLX (66g)
SPI1 (57g)
BATF3 (12g)
ZNF32 (27g)
ZNF274 (129g)
IKZF1 (12g)
TAF7 (1107g)
ZNF66 (69g)
ZBTB7A (8g)
KLF13 (91g)
MYBL1 (54g)
EGR4 (61g)
HOXA9 (36g)
ING4 (8g)
CTBP1 (6g)
TFEB (66g)
EOMES (136g)
CEBPD (105g)
ZEB2 (8g)
NFIL3 (272g)
IRF8 (212g)
TBX21 (213g)
Regulon activity
2
1
0
−1
−2
CD4−CCR7
CD4−CXCR6
CD4−FOXP3
CD4−NR4A3
CD4−c9
CD4−c10
CD8−GZMK
CD8−KLRD1
CD8−KLRD1−CCL4L2
CD8−ZNF683
CD8−CXCL13
D
TCF7 (35g)
LEF1 (40g)
RUNX2 (47g)
ELK3 (312g)
RORA (201g)
FOXO1 (170g)
NFKB2 (413g)
FOXP1 (173g)
ETV6 (127g)
NFKB1 (447g)
BACH1 (206g)
BATF (230g)
FOXP3 (105g)
IKZF2 (6g)
CREB3L2 (183g)
STAT3 (14g)
HLF (20g)
NFATC1 (9g)
IRF5 (27g)
HEYL (3g)
SIRT6 (39g)
MLX (66g)
STAT5A (20g)
SMAD1 (23g)
BATF3 (12g)
SPI1 (57g)
ZNF32 (27g)
ZNF274 (129g)
TFE3 (36g)
BHLHE41 (41g)
EGR4 (61g)
HOXA9 (36g)
ATF3 (517g)
EGR1 (191g)
IKZF1 (12g)
TAF7 (1107g)
ZNF66 (69g)
ZBTB7A (8g)
KLF13 (91g)
MYBL1 (54g)
ETV1 (300g)
EOMES (136g)
CTBP1 (6g)
TFEB (66g)
ING4 (8g)
CEBPD (105g)
ZEB2 (8g)
NFIL3 (272g)
TBX21 (213g)
IRF8 (212g)
Regulon activity
2
1
0
−1
−2
CD4−CCR7.NC
CD4−CCR7.AIS
CD4−CCR7.MIA
CD4−CCR7.IA
CD4−CXCR6.NC
CD4−CXCR6.AIS
CD4−CXCR6.MIA
CD4−CXCR6.IA
CD4−FOXP3.NC
CD4−FOXP3.AIS
CD4−FOXP3.MIA
CD4−FOXP3.IA
CD4−NR4A3.AIS
CD4−NR4A3.MIA
CD4−NR4A3.IA
CD4−c9.NC
CD4−c9.AIS
CD4−c9.MIA
CD4−c9.IA
CD4−c10.NC
CD4−c10.AIS
CD4−c10.MIA
CD4−c10.IA
CD8−GZMK.NC
CD8−GZMK.AIS
CD8−GZMK.MIA
CD8−GZMK.IA
CD8−KLRD1.NC
CD8−KLRD1.MIA
CD8−KLRD1.IA
CD8−KLRD1−CCL4L2.NC
CD8−KLRD1−CCL4L2.AIS
CD8−KLRD1−CCL4L2.MIA
CD8−KLRD1−CCL4L2.IA
CD8−ZNF683.NC
CD8−ZNF683.AIS
CD8−ZNF683.MIA
CD8−ZNF683.IA
CD8−CXCL13.IA

Figure S4 Analysis of the cellular composition of T cells. A: Relative fractions of eleven major T cell subtypes of the participants. B: Comparison of the densities for $NR4A3^+$ (top left) or $FOXP3^+$ (bottom left) cells among different pathological stages of LUAD (two–sided Wilcoxon rank–sum test, *$p<0.05$, **$p<0.01$) with representative IHC images showing on the right. The counts of five randomly chosen 1 mm^2 areas from each sample were recorded for comparison. Each group included counts from 5 cases with 25 areas. Scale bar, 50 µm. C–D: Heatmap showing the regulation of gene expressions by transcription factors in different cell types (C) and tumor stages (D) for the T cells using SCENIC.

Figure S5

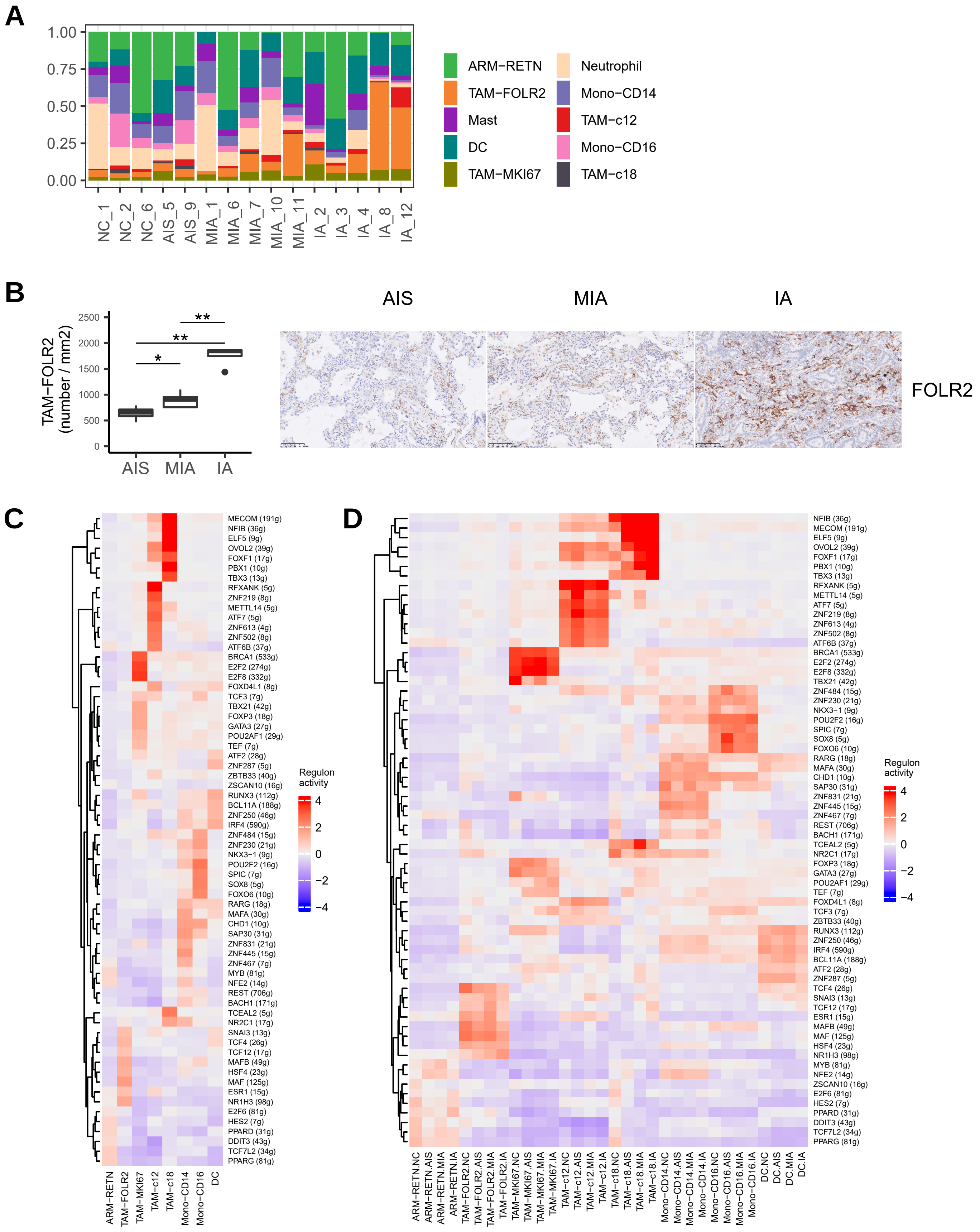
A
1.00
0.75
0.50
0.25
0.00
NC_1
NC_2
NC_6
AIS_5
AIS_9
MIA_1
MIA_6
MIA_7
MIA_10
MIA_11
IA_2
IA_3
IA_4
IA_8
IA_12
ARM−RETN
TAM−FOLR2
Mast
DC
TAM−MKI67
Neutrophil
Mono−CD14
TAM−c12
Mono−CD16
TAM−c18
B
TAM−FOLR2 (number / mm2)
2500
2000
1500
1000
500
0
AIS
MIA
IA
*
**
**
AIS
MIA
IA
FOLR2
C
MECOM (191g)
NFIB (36g)
ELF5 (9g)
OVOL2 (39g)
FOXF1 (17g)
PBX1 (10g)
TBX3 (13g)
RFXANK (5g)
ZNF219 (8g)
METTL14 (5g)
ATF7 (5g)
ZNF613 (4g)
ZNF502 (8g)
ATF6B (37g)
BRCA1 (533g)
E2F2 (274g)
E2F8 (332g)
FOXD4L1 (8g)
TCF3 (7g)
TBX21 (42g)
FOXP3 (18g)
GATA3 (27g)
POU2AF1 (29g)
TEF (7g)
ATF2 (28g)
ZNF287 (5g)
ZBTB33 (40g)
ZSCAN10 (16g)
RUNX3 (112g)
BCL11A (188g)
ZNF250 (46g)
IRF4 (590g)
ZNF484 (15g)
ZNF230 (21g)
NKX3−1 (9g)
POU2F2 (16g)
SPIC (7g)
SOX8 (5g)
FOXO6 (10g)
RARG (18g)
MAFA (30g)
CHD1 (10g)
SAP30 (31g)
ZNF831 (21g)
ZNF445 (15g)
ZNF467 (7g)
MYB (81g)
NFE2 (14g)
REST (706g)
BACH1 (171g)
TCEAL2 (5g)
NR2C1 (17g)
SNAI3 (13g)
TCF4 (26g)
TCF12 (17g)
MAFB (49g)
HSF4 (23g)
MAF (125g)
ESR1 (15g)
NR1H3 (98g)
E2F6 (81g)
HES2 (7g)
PPARD (31g)
DDIT3 (43g)
TCF7L2 (34g)
PPARG (81g)
ARM−RETN
TAM−FOLR2
TAM−MKI67
TAM−c12
TAM−c18
Mono−CD14
Mono−CD16
DC
Regulon activity
4
2
0
−2
−4
D
NFIB (36g)
MECOM (191g)
ELF5 (9g)
OVOL2 (39g)
FOXF1 (17g)
PBX1 (10g)
TBX3 (13g)
RFXANK (5g)
METTL14 (5g)
ATF7 (5g)
ZNF219 (8g)
ZNF613 (4g)
ZNF502 (8g)
ATF6B (37g)
BRCA1 (533g)
E2F2 (274g)
E2F8 (332g)
TBX21 (42g)
ZNF484 (15g)
ZNF230 (21g)
NKX3−1 (9g)
POU2F2 (16g)
SPIC (7g)
SOX8 (5g)
FOXO6 (10g)
RARG (18g)
MAFA (30g)
CHD1 (10g)
SAP30 (31g)
ZNF831 (21g)
ZNF445 (15g)
ZNF467 (7g)
REST (706g)
BACH1 (171g)
TCEAL2 (5g)
NR2C1 (17g)
FOXP3 (18g)
GATA3 (27g)
POU2AF1 (29g)
TEF (7g)
FOXD4L1 (8g)
TCF3 (7g)
ZBTB33 (40g)
RUNX3 (112g)
ZNF250 (46g)
IRF4 (590g)
BCL11A (188g)
ATF2 (28g)
ZNF287 (5g)
TCF4 (26g)
SNAI3 (13g)
TCF12 (17g)
ESR1 (15g)
MAFB (49g)
MAF (125g)
HSF4 (23g)
NR1H3 (98g)
MYB (81g)
NFE2 (14g)
ZSCAN10 (16g)
E2F6 (81g)
HES2 (7g)
PPARD (31g)
DDIT3 (43g)
TCF7L2 (34g)
PPARG (81g)
Regulon activity
4
2
0
−2
−4

Figure S5 Analysis of the cellular composition of myeloid cells.

A: Relative fractions of ten major myeloid cell subtypes of the participants.
B: Comparison of the densities for FOLR2$^+$ (left) cells among different pathological stages of LUAD (two-sided Wilcoxon rank–sum test, *$p<0.05$, **$p<0.01$) with representative IHC images showing on the right. The number of FOLR2$^+$ cells was evaluated at five randomly chosen identical areas within the neoplastic region of each sample. The density of each area was calculated by dividing the number of positive cells by the area (mm^2) of the viewed fields. The five values of density for each sample were recorded for comparison. Each group included values from 5 cases with 25 areas. Scale bar, 100 µm. C-D: Heatmap showing the regulation of gene expression by transcription factors in different cell types (C) and tumor stages (D) for the myeloid cells using SCENIC.

Figure S6

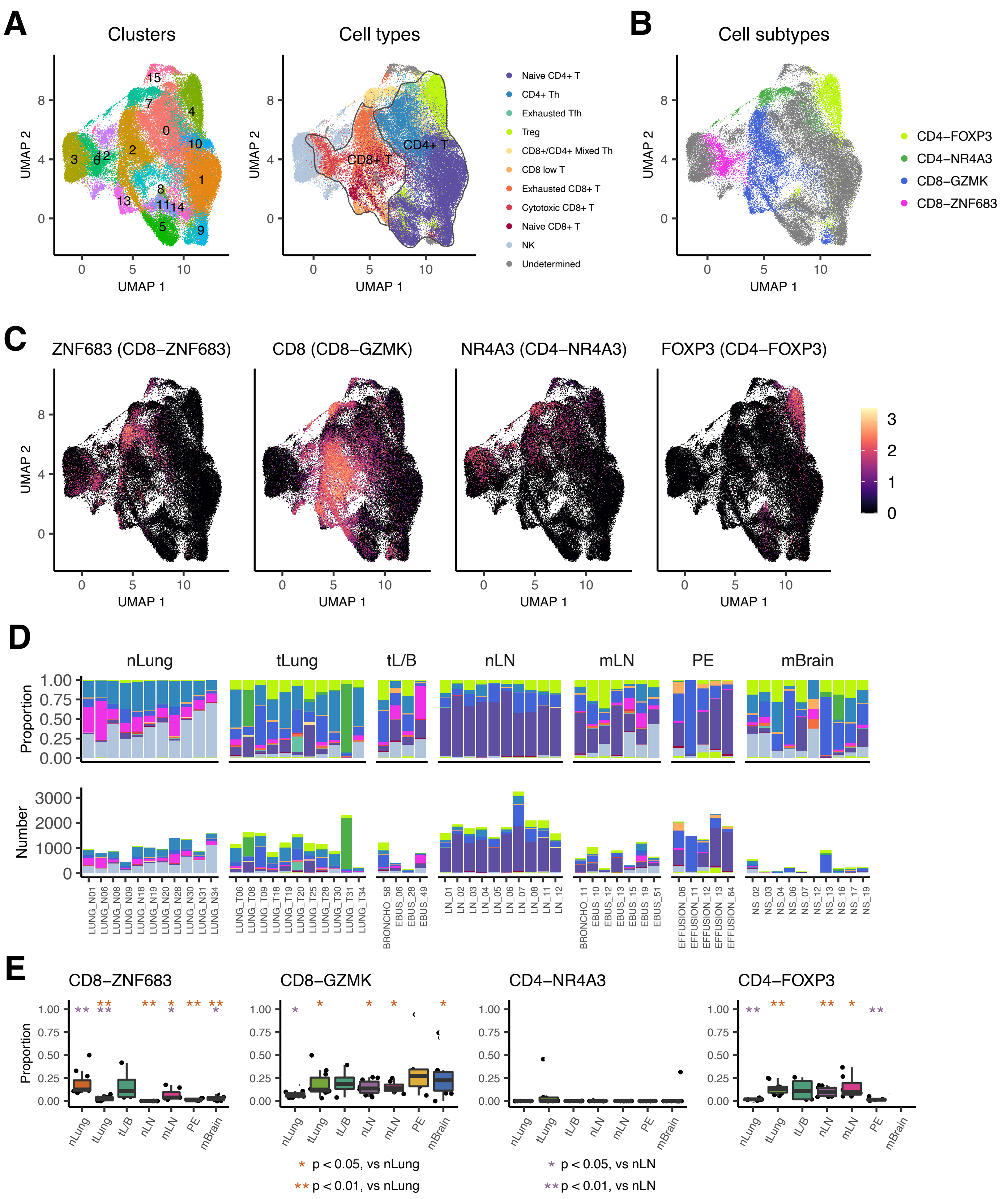

A
Clusters
Cell types
Naive CD4+ T
CD4+ Th
Exhausted Tfh
Treg
CD8+/CD4+ Mixed Th
CD8 low T
Exhausted CD8+ T
Cytotoxic CD8+ T
Naive CD8+ T
NK
Undetermined
B
Cell subtypes
CD4−FOXP3
CD4−NR4A3
CD8−GZMK
CD8−ZNF683
UMAP 1
UMAP 2
C
ZNF683 (CD8−ZNF683)
CD8 (CD8−GZMK)
NR4A3 (CD4−NR4A3)
FOXP3 (CD4−FOXP3)
D
nLung
tLung
tL/B
nLN
mLN
PE
mBrain
Proportion
Number
E
CD8−ZNF683
CD8−GZMK
CD4−NR4A3
CD4−FOXP3
* p < 0.05, vs nLung
** p < 0.01, vs nLung
* p < 0.05, vs nLN
** p < 0.01, vs nLN

Figure S6 Analysis of T cell subtypes from normal tissues to early and advanced stages of LUAD using Kim and colleagues' single−cell dataset (PMID: 32385277). A: UMAP plot of T cells, color−coded by clusters (left) or Kim's original cell types (right). B: UMAP plot of CD4−FOXP3, CD4−NR4A3, CD8−GZMK, and CD8−ZNF683 subsets identified by the specific markers in our study. C: UMAP plot of selected marker gene expressions for cell subsets showing in B. D: Relative proportion (top panel) or cell number (bottom panel) of T cell subsets in each sample among different types of lung cancer samples. nLung, normal lung tissues, n=11; tLung, cancer tissues from primary sites, n=11; tL/B, cancer tissues from biopsies or metastases, n=4; nLN, normal lymph nodes, n=10; mLN, metastatic lymph nodes, n=7; PE, pleural fluids, n=5; mBrain, brain metastases, n=10. Colors represent assigned cell types as in A (right) and B. E: Tissue preference of CD8−ZNF683, CD8−GZMK, CD4−NR4A3, and CD4−FOXP3 cell types. Black dots represent different patients. *$p<0.05$; **$p<0.01$, two−sided Wilcoxon rank−sum test.

Figure S7

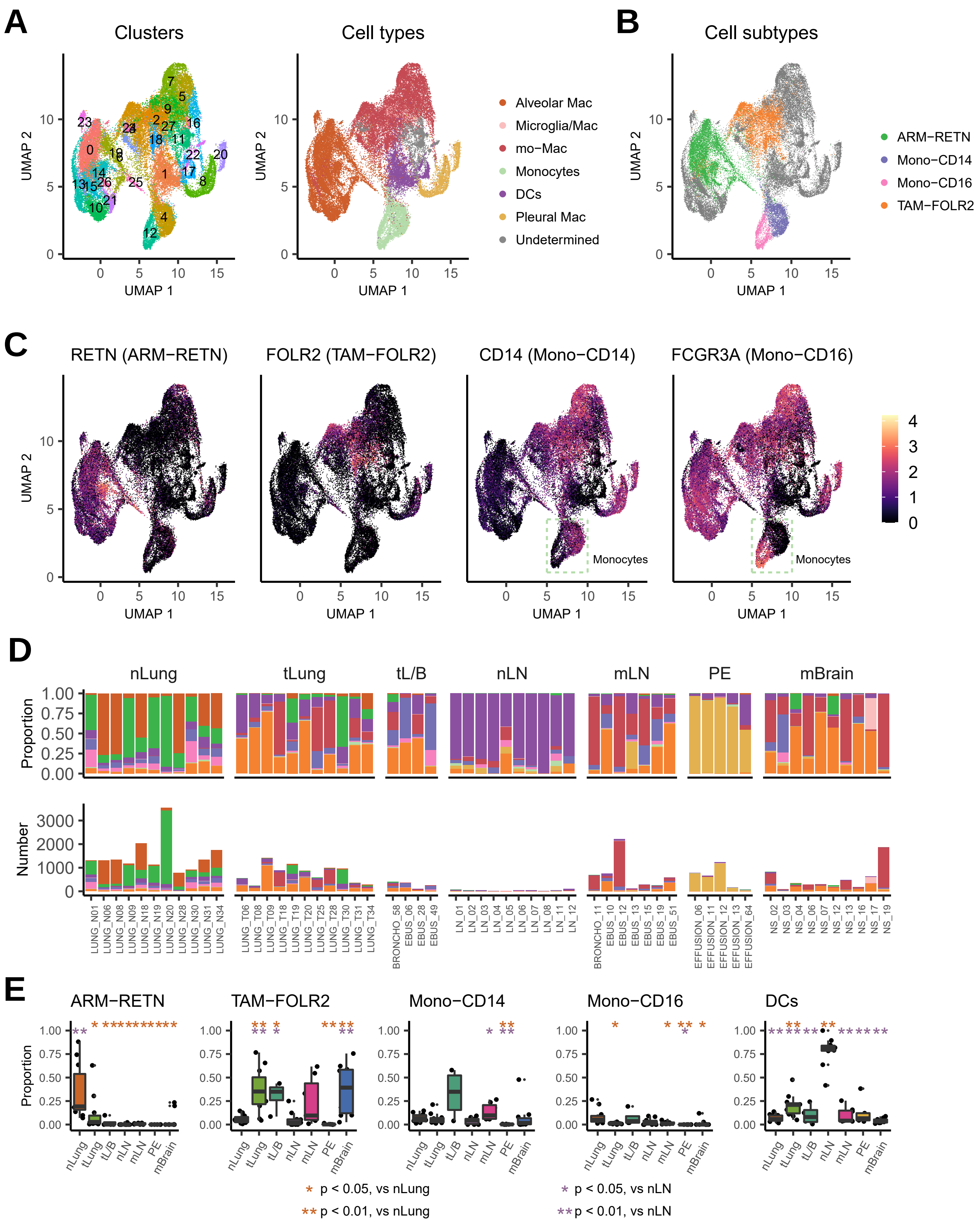

A
Clusters
Cell types
Alveolar Mac
Microglia/Mac
mo−Mac
Monocytes
DCs
Pleural Mac
Undetermined
B
Cell subtypes
ARM−RETN
Mono−CD14
Mono−CD16
TAM−FOLR2
UMAP 1
UMAP 2
C
RETN (ARM−RETN)
FOLR2 (TAM−FOLR2)
CD14 (Mono−CD14)
FCGR3A (Mono−CD16)
Monocytes
D
nLung
tLung
tL/B
nLN
mLN
PE
mBrain
Proportion
Number
E
ARM−RETN
TAM−FOLR2
Mono−CD14
Mono−CD16
DCs
Proportion
* p < 0.05, vs nLung
** p < 0.01, vs nLung
* p < 0.05, vs nLN
** p < 0.01, vs nLN

Figure S7 Analysis of myeloid cell subtypes from normal tissues to early and advanced stages of LUAD using Kim and colleagues' single−cell dataset (PMID: 32385277). A: UMAP plot of myeloid cells, color−coded by clusters (left) and Kim's original cell types (right). B: UMAP plot of ARM−RETN, TAM−FOLR2, Mono−CD14, and Mono−CD16 subsets identified by the specific markers in our study. C: UMAP plot of selected marker gene expressions for cell subsets showing in B. D: Cell relative proportion (top panel) or cell number (bottom panel) of myeloid subsets in each sample among different types of lung cancer samples. nLung, normal lung tissues, n=11; tLung, cancer tissues from primary sites, n=11; tL/B, cancer tissues from biopsies or metastases, n=4; nLN, normal lymph nodes, n=10; mLN, metastatic lymph nodes, n=7; PE, pleural fluids, n=5; mBrain, brain metastases, n=10. Colors represent assigned cell types as in A (right) and B. E: Tissue preference of ARM−RETN, TAM−FOLR2, Mono−CD14, Mono−CD16, and DCs cell types. Black dots represent different patients. $^*p<0.05$; $^{**}p<0.01$, two−sided Wilcoxon rank−sum test.

Figure S8

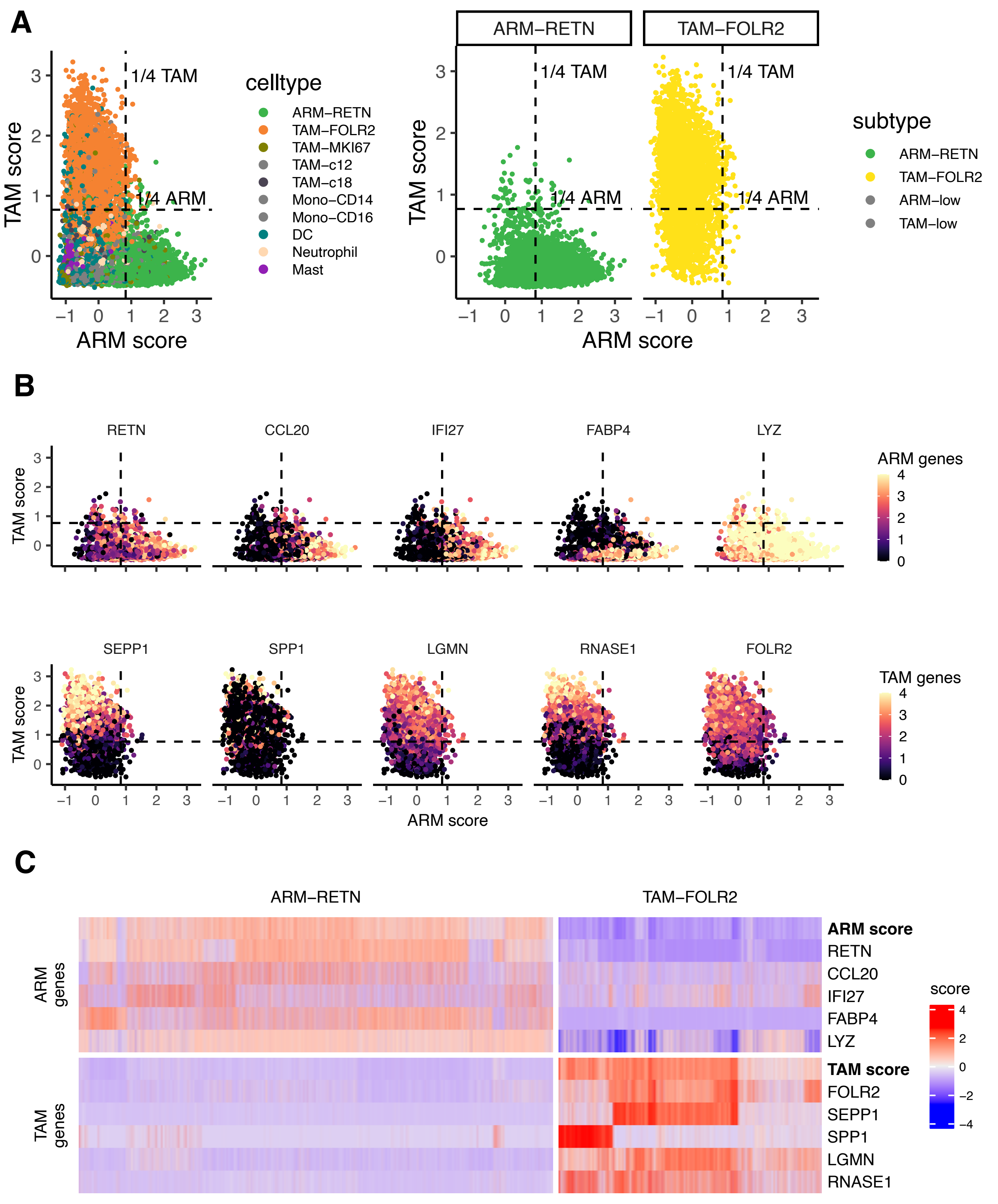

Figure S8 Principal coordinate analysis of different myeloid cell subtypes. A: Two−dimensional visualization based on TAM and ARM signatures of myeloid cells (left) and two major ARM and TAM subtypes (right). B: Two−dimensional visualization of ARM or TAM specifically expressed genes. C:Heatmap of TAM and ARM signature scores and expressions of signature genes in ARM−RETN (left) or TAM−FOLR2 (right) cells.

Figure S9

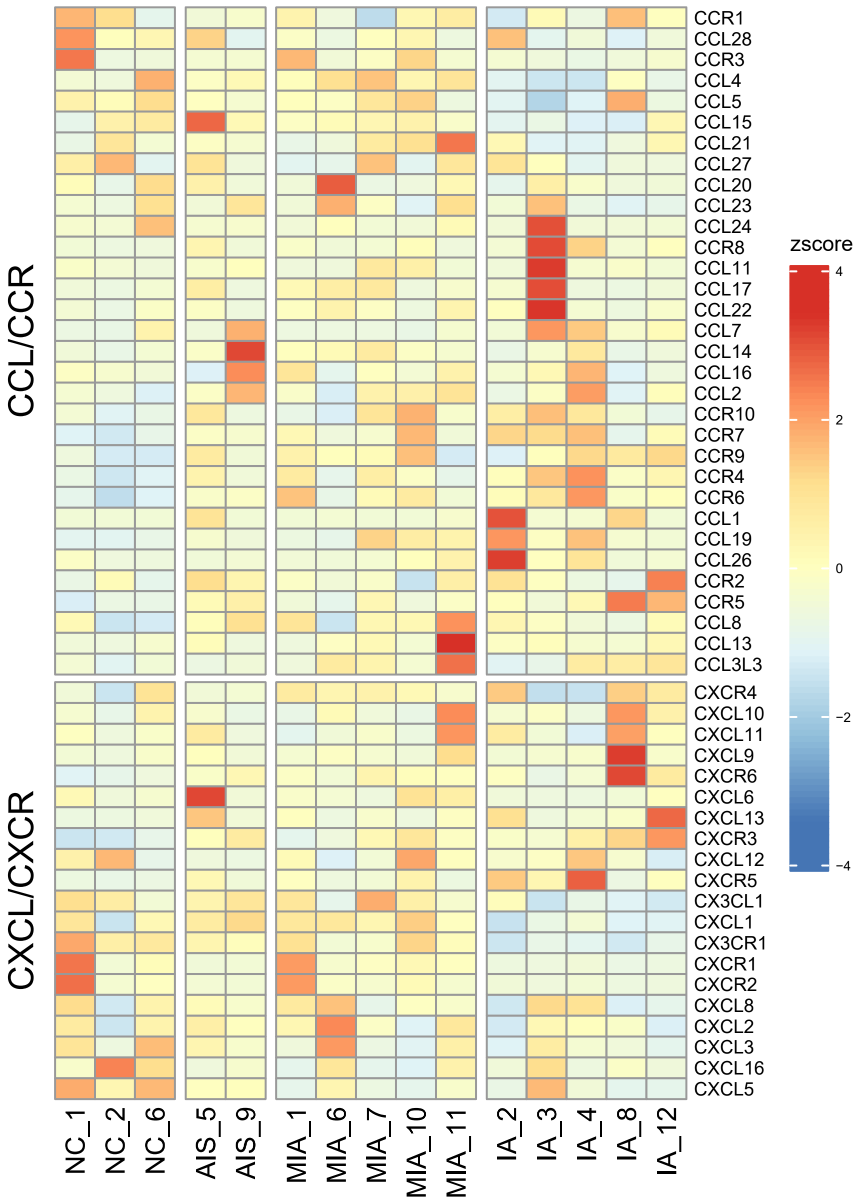

Figure S9 Heatmap of the chemokine ligand/receptor transcriptome of each participant.

Figure S10

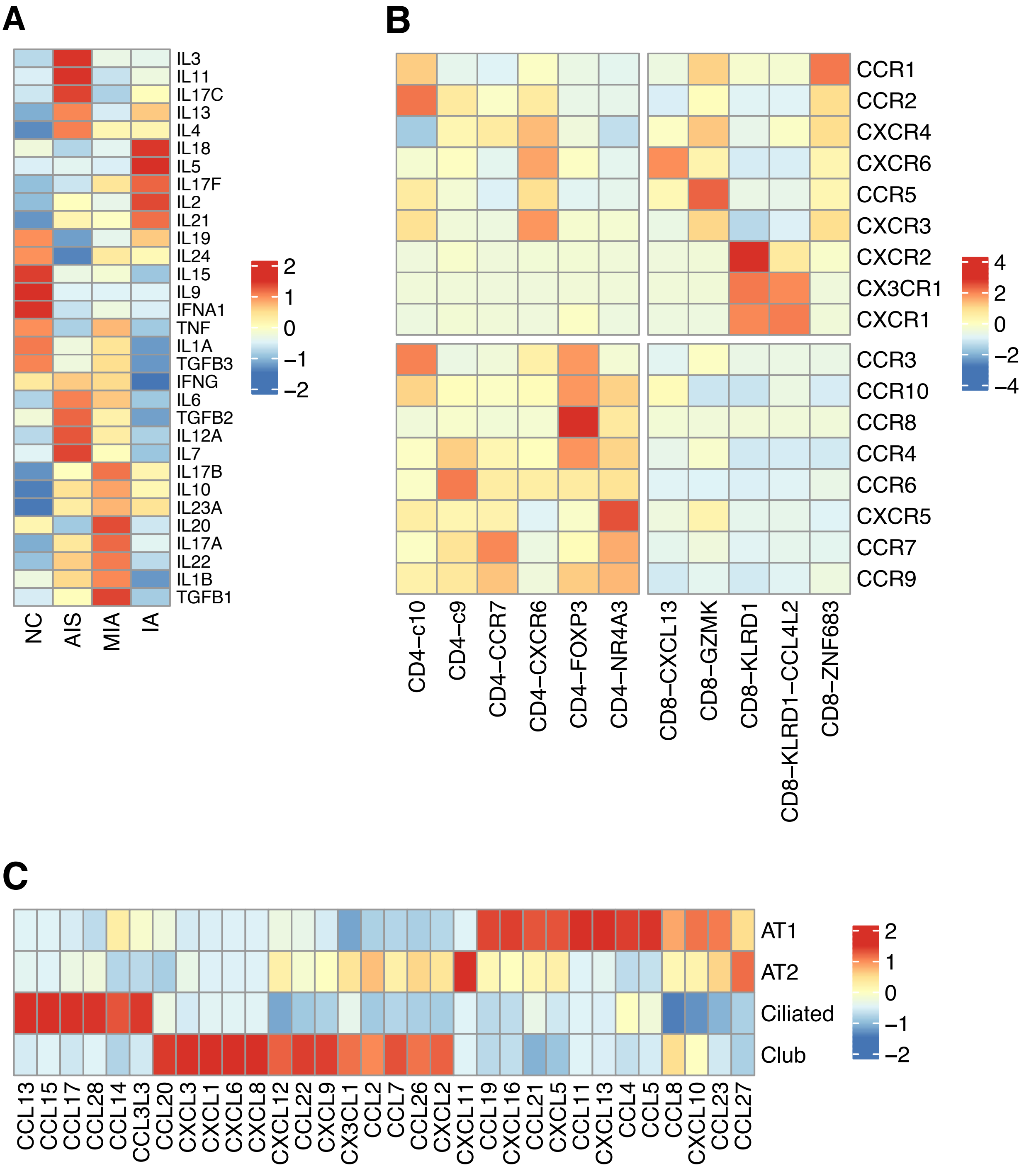

Figure S10 Heatmap of the relative expressions of chemokine ligand/receptor transcriptomes. A: Heatmap of the expression levels of inflammatory factor transcriptomes in normal lung samples and lung tumors at different pathological stages. B: Heatmap of the expression levels of chemokine receptors (CCRs/CXCRs) in T cells. C: Heatmap of the expression levels of chemokine ligands in epithelial cells.

Figure S11

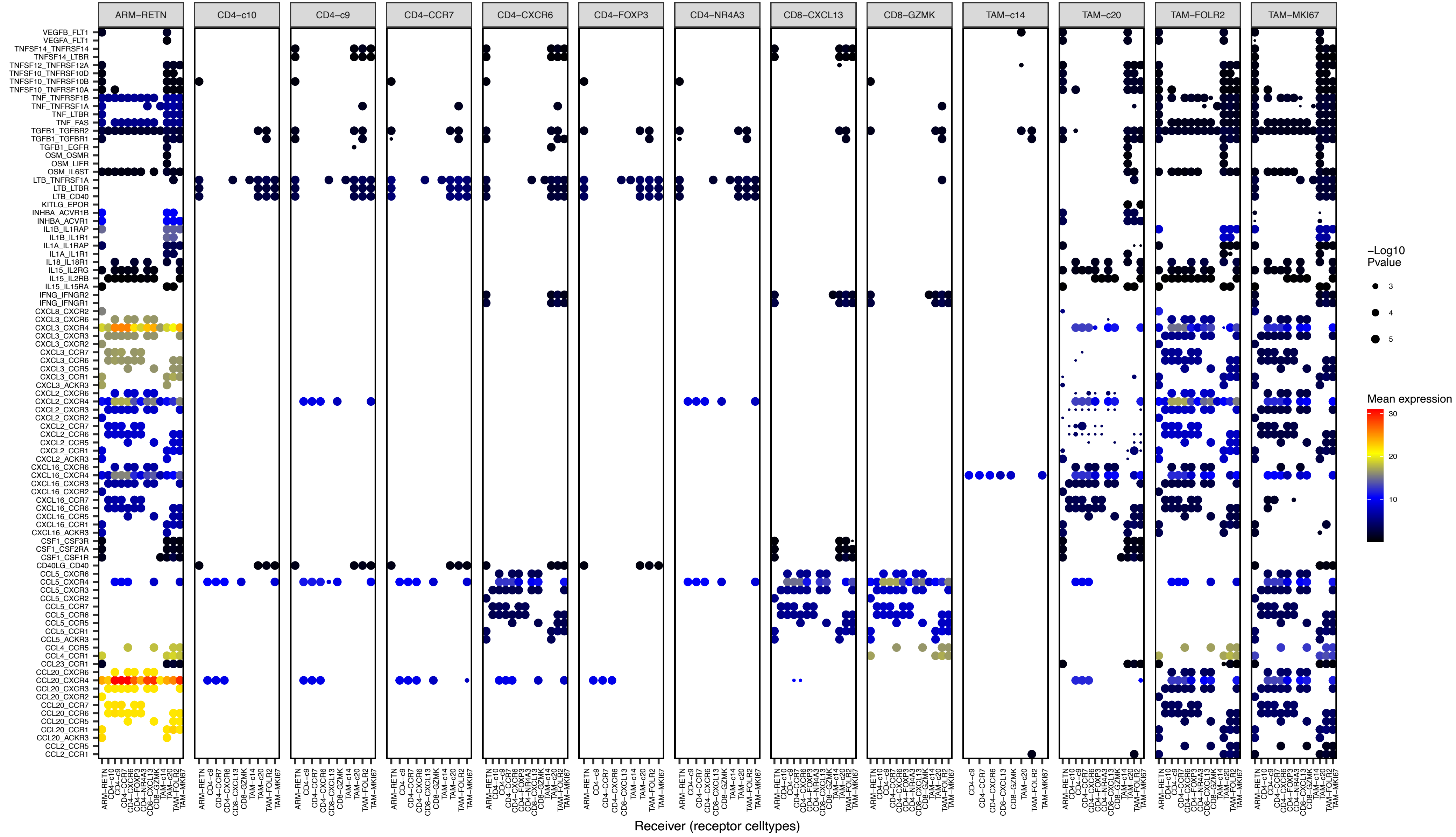

Figure S11 Overview of the chemokine ligand–receptor interactions. The *p* values (two–tailed permutation test) are indicated by circle size; the scale is on the right. The means of the average expression level of interacting molecule 1 in cluster 1 and interacting molecule 2 in cluster 2 are indicated by color.

Figure S12

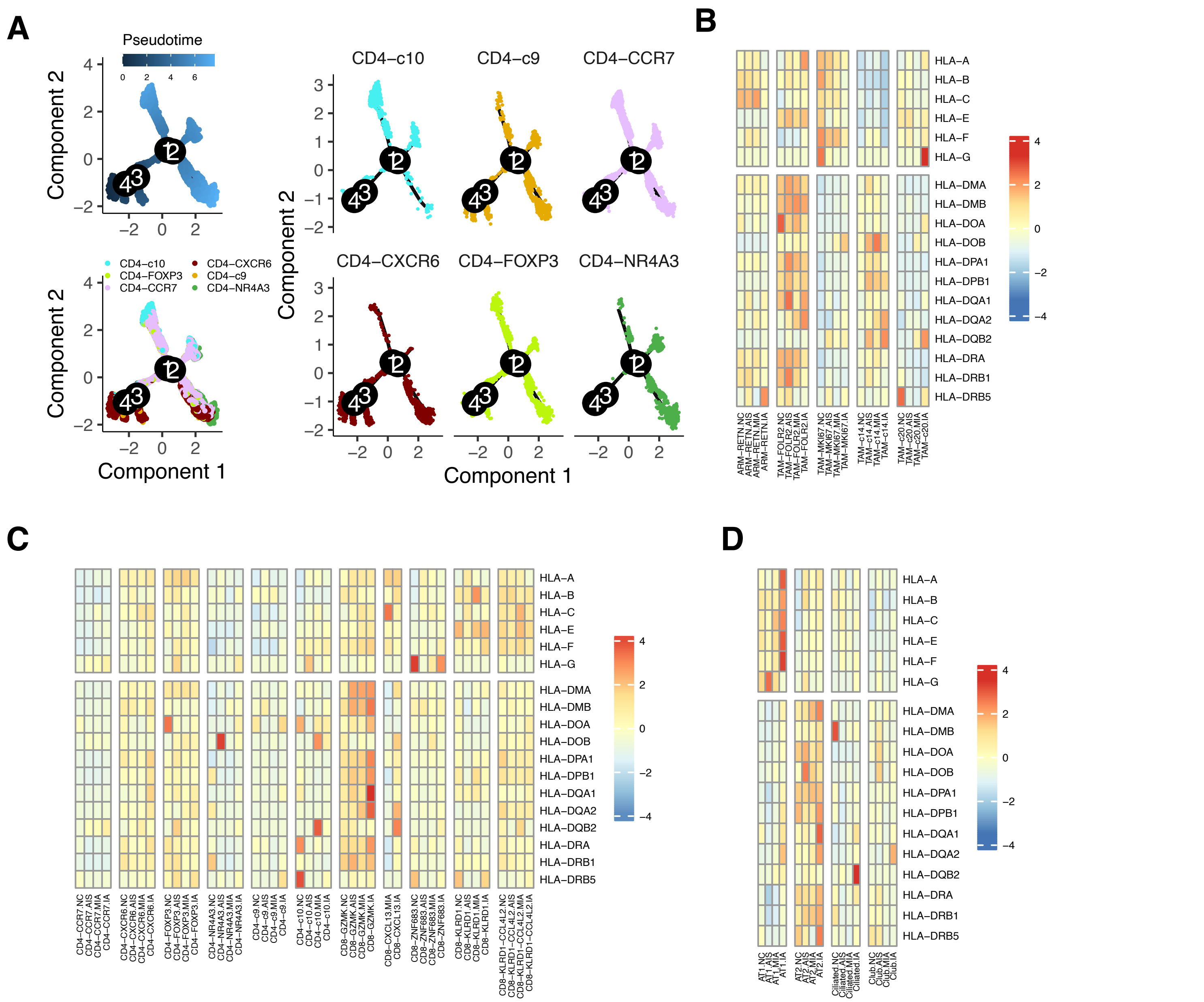

Figure S12 Trajectory analysis of CD4⁺ T cells, and relative expressions of MHC transcriptomes in different cell subtypes. A: Trajectory analysis of CD4⁺ T cell subtypes, in combination (left panel) and separately (right panel) colored by pseudotime or cell subtypes. B–D: Heatmaps of relative expressions of MHC–I (upper panel) and MHC–II (lower panel) genes in different macrophage cell subtypes (B), T cell subtypes (C), and epithelial cell subtypes (D).
