# Supplementary material for: Single-cell profiling reveals the trajectory of FOLR2-expressing tumor-associated macrophages to regulatory T cells in the progression of lung adenocarcinoma
Source: Cell Death Dis. 2023 Aug 2;14(8):493. doi: 10.1038/s41419-023-06021-6 (PMC10397223; doi:10.1038/s41419-023-06021-6)
Supplement: Supplementary file 2 — Supplementary Tables [file 41419_2023_6021_MOESM2_ESM.pdf]

Table S1. Clinical features of 12 LUAD patients involved in the scRNA-seq of this study.

Patient ID	Histological Type[a]	Morphologic pattern	Tumor location[b]	Tumor size (cm)	Gender	Age	Smoking	pTNM	Stage	Driver mutation[c]	PD-L1 (TPS[d])	CD8 staining	TME classification[e]
P1	MIA	lepidic	LLL	1.0	Female	62	No	TiaN0M0	IA1	*EGFR* L858R	<1%	+	III, tolerance
P2	IA	acinar, papillary, micropapillary	RML	1.5	Male	58	Yes	T1bN0M0	IA2	*BRAF* V600X	<1%	+	III, tolerance
P3	IA	papillary, acinar, lepidic	LUL	3.8	Female	60	No	T2aN0M0	IB	*EGFR* G719A	<1%	+	III, tolerance
P4	IA	solid, acinar, papillary, micropapillary	LUL	1.1	Male	63	No	T1bN2M0	IIIA	*CD74-ROS1*(C6:R34)	<1%	+	III, tolerance
P5	AIS	lepidic	LLL	1.0	Female	66	No	TisN0M0	0	*EGFR* L858R	<1%	+	III, tolerance
P6	MIA	lepidic	LUL	1.1	Male	59	No	TiaN0M0	IA1	NA	<1%	+	III, tolerance
P7	MIA	lepidic	RUL	1.3	Female	50	No	TiaN0M0	IA1	*EGFR* 19del	<1%	+	III, tolerance
P8	IA	lepidic, papillary, acinar	LUL	3.1	Female	64	No	T2aN0M0	IB	*EGFR* 19del	<1%	+	III, tolerance
P9	AIS	lepidic	LUL	1.0	Female	38	No	TisN0M0	0	NA	<1%	+	III, tolerance
P10	MIA	lepidic	RLL	1.0	Female	63	No	TiaN0M0	IA1	*EGFR* 20ins	<1%	+	III, tolerance
P11	MIA	lepidic	LUL	1.8	Female	75	No	TiaN0M0	IA1	*EGFR* L858R	<1%	+	III, tolerance
P12	IA	acinar, lepidic	LUL	2.5	Female	54	No	T1cN0M0	IA3	*EGFR* L858R	<1%	+	III, tolerance

a: AIS, adenocarcinoma in situ; MIA, minimally invasive adenocarcinoma; IA, invasive adenocarcinoma;

b: LLL, left lower lobe; LUL, left upper lobe; RML, right middle lobe; RUL, right upper lober; RLL, right lower lobe;

c: NA denotes detection for gene mutations not performed.

d: TPS, tumor proportion score.

e: Referring to Kim, T.K., et al., 2022. (PMID: 35701637)

Table S2. Summary of 10x genomics scRNA-seq data.

Sample ID	Patient ID	Estimated Number of Cells	Mean Reads per Cell	Median Genes per Cell	Number of Reads	Valid Barcodes	Sequencing Saturation	Q30 Bases in Barcode	Q30 Bases in RNA Read	Q30 Bases in UMI	Reads Mapped to Genome
NC_1	P1	7,525	74,243	1,828	558,682,362	96.90%	68.30%	94.10%	90.00%	93.50%	94.70%
MIA_1	P1	7,846	63,896	1,695	501,333,637	97.20%	66.10%	94.30%	90.20%	93.70%	94.50%
NC_2	P2	14,206	36,149	1,707	513,535,824	97.40%	45.50%	94.30%	90.50%	93.70%	94.40%
IA_2	P2	7,567	72,809	1,653	550,946,592	97.70%	63.70%	94.30%	90.10%	93.70%	94.70%
IA_3	P3	9,623	53,333	1,448	513,226,577	97.70%	43.70%	96.20%	92.00%	95.40%	94.60%
IA_4	P4	7,378	70,932	1,483	523,342,506	97.40%	47.00%	96.20%	92.00%	95.50%	95.60%
AIS_5	P5	9,172	55,451	1,754	508,603,001	98.40%	56.70%	96.20%	90.00%	96.00%	94.10%
NC_6	P6	10,026	54,559	1,439	547,017,116	98.40%	64.20%	96.30%	91.20%	96.10%	95.00%
MIA_6	P6	12,792	41,668	1,943	533,022,264	98.40%	52.50%	96.30%	91.00%	96.10%	95.40%
MIA_7	P7	9,783	54,519	1,671	533,367,842	98.20%	60.20%	97.00%	93.10%	96.90%	95.60%
IA_8	P8	8,459	66,558	1,898	563,017,785	98.20%	62.80%	96.90%	93.20%	96.80%	96.10%
AIS_9	P9	14,470	37,399	1,321	541,174,263	98.10%	65.00%	95.40%	91.30%	94.20%	96.10%
MIA_10	P10	9,053	56,940	1,509	515,484,301	98.10%	73.90%	97.00%	92.90%	96.70%	96.20%
MIA_11	P11	7,070	71,358	2,466	504,507,123	98.20%	62.10%	97.00%	93.20%	96.80%	96.20%
IA_12	P12	9,806	53,092	1,323	520,628,946	98.20%	66.70%	97.00%	93.10%	96.80%	96.30%

Table S2 continued

Sample ID	Patient ID	Reads Mapped Confidently to Genome	Reads Mapped Confidently to Intergenic Regions	Reads Mapped Confidently to Intronic Regions	Reads Mapped Confidently to Exonic Regions	Reads Mapped Confidently to Transcriptome	Reads Mapped Antisense to Gene	Fraction Reads in Cells	Total Genes Detected	Median UMI Counts per Cell
NC_1	P1	91.20%	6.80%	37.80%	46.60%	42.40%	1.90%	84.80%	24,354	4,691
MIA_1	P1	92.20%	7.30%	39.70%	45.20%	41.20%	1.70%	83.70%	24,644	4,458
NC_2	P2	91.20%	8.30%	30.70%	52.10%	47.80%	1.90%	90.90%	24,884	3,859
IA_2	P2	92.30%	7.70%	27.90%	56.70%	53.10%	1.30%	94.00%	24,688	5,089
IA_3	P3	91.80%	11.60%	25.90%	54.30%	50.80%	1.20%	87.70%	25,267	4,233
IA_4	P4	93.10%	9.90%	28.80%	54.30%	50.50%	1.50%	92.70%	25,410	4,346
AIS_5	P5	91.30%	8.70%	24.40%	58.20%	54.40%	1.20%	83.10%	24,787	5,593
NC_6	P6	92.50%	7.60%	21.10%	63.80%	59.90%	1.30%	91.90%	24,169	4,042
MIA_6	P6	93.00%	8.40%	25.30%	59.30%	55.60%	1.30%	92.70%	24,655	5,874
MIA_7	P7	92.00%	9.00%	24.60%	58.30%	55.00%	1.00%	90.10%	24,490	5,242
IA_8	P8	92.60%	6.20%	20.20%	66.30%	62.70%	1.00%	94.70%	24,036	6,543
AIS_9	P9	92.40%	9.10%	21.50%	61.90%	58.10%	1.30%	90.70%	24,371	3,679
MIA_10	P10	93.10%	6.00%	18.80%	68.30%	64.10%	1.40%	81.60%	24,177	4,365
MIA_11	P11	93.40%	8.70%	21.50%	63.20%	59.50%	1.30%	92.40%	24,250	7,781
IA_12	P12	93.50%	7.50%	17.50%	68.50%	64.50%	1.20%	90.00%	24,270	4,577

Table S3. Canonical markers used to annotate each cell type.

Celltype	Sub celltype	Canonical markers		
Epithelial	-	EPCAM	KRT8	
Epithelial	AT1	AGER	CLIC5	
Epithelial	AT2	SFTPB	MUC1	
Epithelial	Ciliated	TPPP3	C20orf85	
Epithelial	Club	SCGB1A1		
T cells	-	CD3D	CD3E	
T cells	$CD4^+$ T cells	CD4		
T cells	$CD4^+$ CCR7	CCR7		
T cells	$CD4^+$ CXCR6	CXCR6		
T cells	$CD4^+$ FOXP3 (Treg)	FOXP3		
T cells	$CD4^+$ NR4A3	NR4A3		
T cells	$CD8^+$ T cells	CD8A	CD8B	
T cells	$CD8^+$ GZMK (Teff)	GZMK		
T cells	$CD8^+$ CXCL13	CXCL13		
T cells	$CD8^+$ ZNF683	ZNF683		
T cells	$CD8^+$ KLRD1	KLRD1		
T cells	$CD8^+$ KLRD1-CCL4L2	KLRD1	CCL4L2	
B cells	-	MS4A1		
B cells	Plasma	JCHAIN		
NK cells	-	NKG7	KLRF1	
Myeloids	-	CD68	MSR1	
Myeloids	Macropahges	MRC1	C1QA	APOE
Myeloids	ARM	FABP4	MCEMP1	MARCO
Myeloids	TAM-FOLR2	FOLR2		
Myeloids	TAM-MIK67	MKI67		
Myeloids	$CD14^+$ monocytes	CD14	VCAN	
Myeloids	$CD16^+$ monocytes	FCGR3A		
Myeloids	Neutrophil	FCGR3B		
Myeloids	Mast	TPSAB1		
Myeloids	DC	CD1C		
Stromal	-	COL1A1	ACTA2	
Endothelial	-	CDH5	VWF	

Table S4. Top 10 significantly differential expressed genes for epithelial cell clusters.

cluster	gene	avg_log2FC	pct.1	pct.2	p_val	p_val_adj
AT2	NAPSA	3.027576059	0.947	0.44	0	0
AT2	NPC2	3.027231195	0.938	0.337	0	0
AT2	SFTPA1	2.854441507	0.668	0.242	0	0
AT2	C4BPA	2.845622961	0.725	0.108	0	0
AT2	SFTPB	2.7250115	0.955	0.44	0	0
AT2	SERPINA1	2.45894219	0.688	0.182	0	0
AT2	SFTPA2	2.904788639	0.686	0.298	1.01E-296	2.56E-292
AT2	SPINK1	2.152811441	0.201	0.03	7.78E-108	1.97E-103
AT2	SFTPC	3.52408706	0.605	0.6	1.63E-09	4.13E-05
AT2	MUC1	1.801458833	0.887	0.416	0	0
Ciliated	C9orf24	4.722751442	0.944	0.022	0	0
Ciliated	C20orf85	4.628528094	0.933	0.013	0	0
Ciliated	C11orf88	4.031508792	0.908	0.006	0	0
Ciliated	RSPH1	4.01873317	0.949	0.041	0	0
Ciliated	C1orf194	4.015958232	0.928	0.01	0	0
Ciliated	SNTN	3.730391019	0.877	0.011	0	0
Ciliated	C5orf49	3.711643971	0.918	0.041	0	0
Ciliated	CAPS	5.643283534	0.99	0.157	1.51E-274	3.83E-270
Ciliated	GSTA1	3.691812941	0.795	0.115	1.93E-207	4.90E-203
Ciliated	TPPP3	3.970365781	0.964	0.243	1.01E-187	2.55E-183
AT1	AGER	3.797691756	0.906	0.38	0	0
AT1	RTKN2	3.371489417	0.781	0.125	0	0
AT1	CAV1	3.222083855	0.92	0.56	0	0
AT1	EMP2	3.060426223	0.923	0.777	0	0
AT1	S100A4	2.802138312	0.807	0.581	0	0
AT1	MT1G	3.906373606	0.246	0.039	6.54E-184	1.66E-179
AT1	MT2A	3.400665946	0.567	0.61	1.31E-41	3.32E-37
AT1	MT1E	3.272162441	0.595	0.675	5.85E-34	1.48E-29
AT1	MT1X	3.696305468	0.377	0.419	5.34E-13	1.35E-08
AT1	CLIC5	2.124860594	0.759	0.353	0	0
Club	BPIFB1	5.916865654	0.637	0.022	0	0
Club	MSMB	4.607143101	0.399	0.006	0	0
Club	CXCL1	3.245080238	0.544	0.063	2.70E-206	6.83E-202
Club	SCGB1A1	8.346279916	0.954	0.338	1.16E-202	2.94E-198
Club	SLPI	3.536651811	0.979	0.637	6.50E-136	1.65E-131
Club	SCGB3A1	3.710658636	0.975	0.507	1.53E-135	3.87E-131
Club	LCN2	3.318209539	0.754	0.227	1.57E-135	3.96E-131
Club	TFF3	3.096110148	0.616	0.134	4.09E-121	1.04E-116
Club	MGP	3.069883528	0.68	0.236	7.14E-83	1.81E-78
Club	SCGB3A2	3.155398816	0.516	0.448	3.09E-08	0.000781934

Table S5. Top 10 significantly differential expressed genes for myeloid clusters.

cluster	gene	avg_log2FC	pct.1	pct.2	p_val	p_val_adj
ARM-RETN	RETN	3.006328226	0.959	0.335	0	0
ARM-RETN	CCL20	2.193048328	0.648	0.213	0	0
ARM-RETN	IFI27	2.178853976	0.521	0.184	0	0
ARM-RETN	FABP4	2.171497789	0.788	0.241	0	0
ARM-RETN	LYZ	2.099935247	0.999	0.807	0	0
ARM-RETN	MARCO	2.093261741	0.978	0.332	0	0
ARM-RETN	INHBA	1.784264933	0.835	0.224	0	0
ARM-RETN	FBP1	1.68836059	0.993	0.527	0	0
ARM-RETN	LPL	1.654510572	0.909	0.27	0	0
ARM-RETN	MCEMP1	1.342202038	0.966	0.263	0	0
DC	CCL17	3.614418936	0.295	0.02	0	0
DC	S100B	2.75663214	0.46	0.064	0	0
DC	GPR183	2.288456636	0.919	0.294	0	0
DC	CD1C	2.114086958	0.597	0.036	0	0
DC	FCER1A	2.097069232	0.514	0.064	0	0
DC	LTB	1.914413462	0.62	0.253	0	0
DC	HLA-DQB2	1.902749759	0.347	0.094	0	0
DC	CLEC10A	1.856974602	0.645	0.121	0	0
DC	CD1E	1.828553224	0.533	0.023	0	0
DC	HLA-DPB1	1.76662412	0.998	0.813	0	0
Mast	TPSB2	7.117379717	0.981	0.033	0	0
Mast	TPSAB1	6.561950138	0.981	0.013	0	0
Mast	CPA3	5.434207547	0.988	0.011	0	0
Mast	MS4A2	4.403679726	0.976	0.007	0	0
Mast	CD69	4.276853431	0.963	0.402	0	0
Mast	HPGD	3.487610427	0.904	0.318	0	0
Mast	HPGDS	3.442615499	0.965	0.318	0	0
Mast	C1orf186	3.330334154	0.916	0.013	0	0
Mast	KIT	3.314534091	0.917	0.008	0	0
Mast	GATA2	3.087340741	0.841	0.007	0	0
Mono-CD14	EREG	3.611849312	0.817	0.205	0	0
Mono-CD14	VCAN	3.433317955	0.875	0.27	0	0
Mono-CD14	FCN1	2.76937259	0.986	0.195	0	0
Mono-CD14	THBS1	2.409535221	0.67	0.365	0	0
Mono-CD14	IL1B	2.361463035	0.856	0.585	0	0
Mono-CD14	TIMP1	2.055915423	0.933	0.678	0	0
Mono-CD14	S100A9	1.897816845	0.984	0.739	0	0
Mono-CD14	NLRP3	1.886006202	0.857	0.318	0	0
Mono-CD14	CD300E	1.77685775	0.743	0.097	0	0

Mono-CD14	CXCL2	1.762294672	0.712	0.431	2.43E-252	6.16E-248
Mono-CD16	CDKN1C	2.628527207	0.791	0.097	0	0
Mono-CD16	LYPD2	2.569816612	0.393	0.005	0	0
Mono-CD16	LILRB2	2.101027351	0.961	0.311	0	0
Mono-CD16	TIMP1	2.094071271	0.978	0.685	0	0
Mono-CD16	FCGR3A	2.085756881	0.973	0.64	0	0
Mono-CD16	MTSS1	1.955370981	0.916	0.147	0	0
Mono-CD16	CD48	1.93658843	0.986	0.452	0	0
Mono-CD16	HES4	1.923887654	0.821	0.129	0	0
Mono-CD16	LST1	1.897203492	1	0.856	0	0
Mono-CD16	POU2F2	1.874959504	0.898	0.22	0	0
Neutrophil	FCGR3B	5.216160191	0.854	0.016	0	0
Neutrophil	G0S2	4.648403089	0.916	0.289	0	0
Neutrophil	IFITM2	4.460400317	0.959	0.613	0	0
Neutrophil	S100A8	4.098121809	0.924	0.552	0	0
Neutrophil	CMTM2	3.894735696	0.556	0.029	0	0
Neutrophil	NAMPT	3.763289605	0.969	0.885	0	0
Neutrophil	PTGS2	3.754686017	0.772	0.408	0	0
Neutrophil	CXCR2	3.717177043	0.543	0.077	0	0
Neutrophil	SLC25A37	3.618983517	0.647	0.351	0	0
Neutrophil	SMCHD1	3.320401893	0.685	0.602	0	0
TAM-c12	AIF1	1.242434352	0.855	0.846	7.97E-75	2.02E-70
TAM-c12	TUBA1B	1.283237297	0.774	0.839	8.59E-50	2.18E-45
TAM-c12	LGALS1	1.17242625	0.811	0.856	1.15E-48	2.90E-44
TAM-c12	FTL	1.217937994	0.995	0.999	6.57E-36	1.66E-31
TAM-c12	SPP1	1.479182342	0.215	0.115	6.56E-16	1.66E-11
TAM-c12	S100B	2.217111069	0.187	0.114	3.09E-10	7.82E-06
TAM-c12	IGHA1	2.053395246	0.093	0.154	0.000605418	1
TAM-c12	IGKC	3.016172256	0.255	0.287	0.352866172	1
TAM-c12	IGLC2	3.54548639	0.124	0.142	0.659106186	1
TAM-c12	SCGB3A1	1.127496813	0.301	0.387	0.85072054	1
TAM-c18	CAV1	2.775892708	0.779	0.058	0	0
TAM-c18	EMP2	2.434911514	0.805	0.124	8.02E-162	2.03E-157
TAM-c18	NAPSA	2.736901588	0.55	0.186	2.06E-41	5.22E-37
TAM-c18	SLPI	2.702781077	0.45	0.121	5.93E-40	1.50E-35
TAM-c18	SFTPB	3.198054973	0.685	0.39	9.79E-27	2.48E-22
TAM-c18	SFTPA2	2.774559596	0.477	0.187	3.23E-23	8.18E-19
TAM-c18	SCGB3A1	4.258639762	0.537	0.384	8.48E-08	0.002147751
TAM-c18	SCGB3A2	3.265786611	0.295	0.162	1.50E-07	0.003809572
TAM-c18	SFTPC	4.131168285	0.644	0.512	0.00028427	1
TAM-c18	SCGB1A1	3.697705214	0.416	0.369	0.45664016	1
TAM-FOLR2	SEPP1	3.544691252	0.793	0.079	0	0
TAM-FOLR2	SPP1	3.152646559	0.415	0.073	0	0
TAM-FOLR2	LGMN	2.926280999	0.923	0.257	0	0
TAM-FOLR2	RNASE1	2.861752955	0.784	0.158	0	0

TAM-FOLR2	APOE	2.554146003	0.921	0.574	0	0
TAM-FOLR2	FOLR2	2.518206142	0.876	0.165	0	0
TAM-FOLR2	A2M	2.469549338	0.966	0.212	0	0
TAM-FOLR2	SLC40A1	2.382171432	0.81	0.106	0	0
TAM-FOLR2	CCL13	2.255573099	0.442	0.03	0	0
TAM-FOLR2	CCL18	2.289413222	0.524	0.28	2.67E-217	6.75E-213
TAM-MKI67	STMN1	2.997634899	0.986	0.506	0	0
TAM-MKI67	HIST1H4C	2.927138902	0.781	0.278	0	0
TAM-MKI67	HMGB2	2.734587559	0.97	0.612	0	0
TAM-MKI67	KIAA0101	2.400344279	0.865	0.021	0	0
TAM-MKI67	TYMS	2.287835888	0.79	0.018	0	0
TAM-MKI67	TUBB	2.233377409	0.949	0.694	0	0
TAM-MKI67	CCL5	2.331661343	0.455	0.17	1.82E-139	4.60E-135
TAM-MKI67	NKG7	2.509363316	0.37	0.127	4.39E-121	1.11E-116
TAM-MKI67	GNLY	2.434969564	0.188	0.077	5.21E-40	1.32E-35
TAM-MKI67	MKI67	2.087943551	0.704	0.008	0	0

Table S6. Top 10 significantly differential expressed genes for T lymphocyte clusters.

cluster	gene	avg_log2FC	pct.1	pct.2	p_val	p_val_adj
CD4-CCR7	KLF2	1.318569604	0.805	0.507	0	0
CD4-CCR7	LTB	1.219432252	0.922	0.532	0	0
CD4-CCR7	CCR7	1.098495163	0.449	0.122	0	0
CD4-CCR7	GPR183	0.901727024	0.783	0.448	0	0
CD4-CCR7	JUNB	0.812783967	0.967	0.85	0	0
CD4-CCR7	LDHB	0.778527789	0.873	0.598	0	0
CD4-CCR7	RPS6	0.743755168	1	0.991	0	0
CD4-CCR7	RPL32	0.731657182	1	0.996	0	0
CD4-CCR7	EEF1B2	0.712710083	0.995	0.927	0	0
CD4-CCR7	NFKBIA	0.72402555	0.878	0.718	3.22E-213	8.15E-209
CD4-CXCR6	CD40LG	1.102078035	0.564	0.205	0	0
CD4-CXCR6	ALOX5AP	1.084450058	0.839	0.521	0	0
CD4-CXCR6	RBPJ	1.074518242	0.634	0.282	0	0
CD4-CXCR6	BATF	1.019363661	0.536	0.276	0	0
CD4-CXCR6	CXCR6	0.935340591	0.415	0.145	0	0
CD4-CXCR6	CD52	0.900850697	0.984	0.906	0	0
CD4-CXCR6	S100A4	0.869610741	0.991	0.914	0	0
CD4-CXCR6	SH3BGRL3	0.809776939	0.989	0.942	0	0
CD4-CXCR6	CAPG	0.795597101	0.486	0.218	0	0
CD4-CXCR6	PLIN2	1.020182879	0.53	0.299	6.65E-262	1.68E-257
CD4-FOXP3	TNFRSF4	2.77156298	0.651	0.118	0	0
CD4-FOXP3	TNFRSF18	2.236753097	0.611	0.104	0	0
CD4-FOXP3	PMAIP1	1.918484491	0.65	0.2	0	0
CD4-FOXP3	TIGIT	1.909787644	0.731	0.173	0	0
CD4-FOXP3	LAIR2	1.833105652	0.511	0.06	0	0
CD4-FOXP3	CTLA4	1.761446025	0.685	0.158	0	0
CD4-FOXP3	PMCH	1.565253623	0.108	0.001	0	0
CD4-FOXP3	ICA1	1.533563162	0.412	0.024	0	0
CD4-FOXP3	CARD16	1.451557765	0.664	0.288	0	0
CD4-FOXP3	FOXP3	1.286707722	0.345	0.012	0	0
CD4-NR4A3	PABPC1	1.487155355	0.95	0.747	0	0
CD4-NR4A3	YPEL5	1.445988882	0.872	0.55	0	0
CD4-NR4A3	ATP1B3	1.405269257	0.683	0.399	0	0
CD4-NR4A3	CREM	1.369125716	0.893	0.577	0	0
CD4-NR4A3	REL	1.367751457	0.742	0.435	0	0
CD4-NR4A3	KDM6B	1.348970729	0.576	0.222	0	0
CD4-NR4A3	LMNA	1.342897089	0.734	0.387	0	0
CD4-NR4A3	HP	1.32372204	0.349	0.017	0	0
CD4-NR4A3	IRS2	1.293879553	0.44	0.112	0	0
CD4-NR4A3	NR4A3	1.236197009	0.443	0.129	0	0
CD4-c9	FKBP5	1.073768729	0.876	0.457	0	0
CD4-c9	XIST	1.15235493	0.757	0.351	1.66E-302	4.21E-298

CD4-c9	AIM1	1.054457161	0.821	0.42	6.47E-269	1.64E-264
CD4-c9	IL7R	1.013126686	0.95	0.729	1.28E-260	3.25E-256
CD4-c9	SC5D	0.93719792	0.559	0.215	7.85E-232	1.99E-227
CD4-c9	CELF2	0.849786084	0.871	0.514	2.57E-226	6.51E-222
CD4-c9	RP11-138A9.1	0.861611009	0.64	0.272	7.95E-222	2.01E-217
CD4-c9	AHNAK	1.031549725	0.911	0.67	3.42E-197	8.65E-193
CD4-c9	ANKRD28	0.85602349	0.643	0.328	3.90E-149	9.87E-145
CD4-c9	KLRB1	0.918293849	0.666	0.376	1.50E-136	3.80E-132
CD4-c10	GIMAP7	0.754990702	0.533	0.435	1.29E-42	3.27E-38
CD4-c10	LTB	0.646361203	0.663	0.597	2.12E-36	5.37E-32
CD4-c10	GIMAP4	0.693093746	0.457	0.356	6.30E-35	1.59E-30
CD4-c10	N4BP2L2	0.636737408	0.678	0.678	1.39E-31	3.52E-27
CD4-c10	NKTR	0.779349543	0.437	0.4	3.90E-18	9.86E-14
CD4-c10	SFTPC	1.353558669	0.272	0.41	2.05E-13	5.20E-09
CD4-c10	DDX17	0.645032798	0.525	0.55	2.20E-11	5.56E-07
CD4-c10	ANKRD36C	0.668849225	0.167	0.129	3.51E-08	0.000888087
CD4-c10	IGKC	2.173717453	0.209	0.29	3.66E-06	0.092655164
CD4-c10	IGHA1	2.221410193	0.084	0.124	0.000113648	1
CD8-CXCL13	CXCL13	3.285524942	0.436	0.012	0	0
CD8-CXCL13	KRT86	2.593839354	0.685	0.008	0	0
CD8-CXCL13	KRT81	1.935788281	0.473	0.003	0	0
CD8-CXCL13	FAM3C	2.073549684	0.679	0.092	5.17E-160	1.31E-155
CD8-CXCL13	CTLA4	2.079241011	0.83	0.194	2.63E-112	6.66E-108
CD8-CXCL13	CTSW	2.04162721	0.879	0.253	1.05E-100	2.65E-96
CD8-CXCL13	AC092580.4	1.908459657	0.788	0.207	7.07E-94	1.79E-89
CD8-CXCL13	CD7	2.249250367	0.939	0.443	1.52E-84	3.85E-80
CD8-CXCL13	RGS1	1.910314125	0.976	0.667	1.86E-70	4.70E-66
CD8-CXCL13	GZMB	1.870274525	0.77	0.247	1.05E-56	2.65E-52
CD8-GZMK	GZMK	3.42387827	0.954	0.161	0	0
CD8-GZMK	CCL4L2	3.032351851	0.612	0.147	0	0
CD8-GZMK	CCL3	2.292251407	0.327	0.089	0	0
CD8-GZMK	CCL4	2.069719195	0.943	0.453	0	0
CD8-GZMK	CCL3L3	1.971815865	0.406	0.057	0	0
CD8-GZMK	GZMA	1.667125534	0.946	0.479	0	0
CD8-GZMK	CST7	1.592735482	0.941	0.469	0	0
CD8-GZMK	DUSP2	1.57388347	0.923	0.682	0	0
CD8-GZMK	CMC1	1.566853821	0.608	0.244	0	0
CD8-GZMK	CRTAM	1.558377987	0.439	0.082	0	0
CD8-KLRD1	GNLY	3.055892031	0.838	0.107	0	0
CD8-KLRD1	KLRD1	2.249034268	0.875	0.187	0	0
CD8-KLRD1	FGFBP2	2.123363443	0.761	0.084	0	0
CD8-KLRD1	ZEB2	1.739782969	0.851	0.239	0	0
CD8-KLRD1	GZMB	1.658839314	0.866	0.213	0	0
CD8-KLRD1	TRGC2	1.655383879	0.689	0.215	0	0
CD8-KLRD1	NKG7	1.632873494	0.996	0.396	0	0

CD8-KLRD1	PRF1	1.599722794	0.836	0.251	0	0
CD8-KLRD1	GZMH	1.528296372	0.914	0.272	0	0
CD8-KLRD1	FCGR3A	1.426458294	0.487	0.074	0	0
CD8-KLRD1-CCL4L2	FGFBP2	2.93486646	0.804	0.058	0	0
CD8-KLRD1-CCL4L2	NKG7	2.778023667	0.998	0.376	0	0
CD8-KLRD1-CCL4L2	GNLY	2.737641966	0.6	0.106	0	0
CD8-KLRD1-CCL4L2	GZMH	2.436962983	0.932	0.249	0	0
CD8-KLRD1-CCL4L2	GZMB	2.01032333	0.791	0.198	0	0
CD8-KLRD1-CCL4L2	KLRD1	1.800063181	0.741	0.178	0	0
CD8-KLRD1-CCL4L2	PRF1	1.735444758	0.821	0.233	0	0
CD8-KLRD1-CCL4L2	FCGR3A	1.618248243	0.486	0.06	0	0
CD8-KLRD1-CCL4L2	CST7	1.534361936	0.969	0.485	0	0
CD8-KLRD1-CCL4L2	CCL4L2	1.103032243	0.481	0.178	2.20E-284	5.56E-280
CD8-ZNF683	KLRC1	1.952568408	0.334	0.025	0	0
CD8-ZNF683	TOB1	1.351531081	0.673	0.397	0	0
CD8-ZNF683	ITGA1	1.34828884	0.413	0.07	0	0
CD8-ZNF683	XCL1	1.340269272	0.377	0.089	0	0
CD8-ZNF683	CD8A	1.182580432	0.634	0.232	0	0
CD8-ZNF683	YBX3	1.024693013	0.361	0.119	0	0
CD8-ZNF683	HSP90AA1	1.022213599	0.961	0.941	0	0
CD8-ZNF683	HSPA1A	1.214270443	0.646	0.426	1.22E-269	3.08E-265
CD8-ZNF683	HSPH1	1.038286568	0.531	0.345	3.55E-210	9.00E-206
CD8-ZNF683	ZNF683	0.905504074	0.271	0.05	0	0

Table S7. IHC validation in an independent cohort (n=15).

	Clinical features of IHC validation samples					IHC evaluation for NR4A3					
IHC validation sample ID	Histological Type[a]	Morphologic pattern	Tumor size (cm)	Gender	Age	NR4A3-area1/mm^2	NR4A3-area2/mm^2	NR4A3-area3/mm^2	NR4A3-area4/mm^2	NR4A3-area5/mm^2	**NR4A3-mean No./mm2**
V1	AIS	lepidic	1.2	Female	36	8	9	1	3	8	5.8
V2	AIS	lepidic	1.5	Female	75	7	2	3	2	3	3.4
V3	AIS	lepidic	1.3	Female	71	2	0	4	1	3	2
V4	AIS	lepidic	1.3	Female	45	11	4	12	3	0	6
V5	AIS	lepidic	1.1	Female	68	8	2	3	4	7	4.8
V6	MIA	lepidic	0.7	Female	49	9	21	4	10	6	10
V7	MIA	lepidic	1.1	Female	51	22	20	29	10	8	17.8
V8	MIA	lepidic	1.3	Female	48	8	13	16	70	10	23.4
V9	MIA	lepidic	1.4	Female	54	5	14	5	2	7	6.6
V10	MIA	lepidic	1.2	Female	60	2	31	4	22	13	14.4
V11	IA	acinar, complex glandular, micropapillary	2.3	Female	59	201	13	44	69	39	73.2
V12	IA	acinar, lepidic, complex glandular	1.8	Female	62	75	62	43	70	61	62.2
V13	IA	cribriform, acinar, micropapillary, complex glandular	1.5	Male	62	34	39	26	30	35	32.8
V14	IA	complex glandular，acinar, solid	2.2	Female	74	9	15	16	5	29	14.8
V15	IA	acinar,papillary, lepidic	2.4	Male	66	38	34	19	45	34	34

Table S7 continued

	IHC evaluation for FOXP3						IHC evaluation for FOLR2					
IHC validation sample ID	FOXP3-area1/mm^2	FOXP3-area2/mm^2	FOXP3-area3/mm^2	FOXP3-area4/mm^2	FOXP3-area5/mm^2	**FOXP3-mean No./mm2**	FOLR2-area1/mm^2	FOLR2-area2/mm^2	FOLR2-area3/mm^2	FOLR2-area4/mm^2	FOLR2-area5/mm^2	**FOLR2-mean No./mm2**
V1	31	62	73	33	44	48.6	743	514	587	477	587	581.6
V2	130	59	101	65	63	83.6	514	624	1101	917	404	712
V3	18	46	11	28	45	29.6	514	881	587	551	734	653.4
V4	38	29	41	41	30	35.8	110	294	917	550	440	462.2
V5	46	47	20	27	29	33.8	771	330	697	1431	734	792.6
V6	64	91	67	113	52	77.4	1468	1064	1027	917	1027	1100.6
V7	45	22	31	85	67	50	844	587	807	587	954	755.8
V8	209	162	118	82	80	130.2	991	1248	440	477	1349	901
V9	73	81	82	136	64	87.2	734	1101	917	514	514	756
V10	81	103	108	123	120	107	954	330	634	1615	1248	956.2
V11	68	162	421	137	309	219.4	2202	2092	1908	1394	1615	1842.2
V12	235	348	346	241	346	303.2	2055	1909	1615	1431	2312	1864.4
V13	201	157	227	237	215	207.4	1505	1358	2129	2276	1468	1747.2
V14	207	238	122	217	183	193.4	1175	1725	2092	1909	2533	1886.8
V15	176	141	132	256	282	197.4	1358	1394	991	1872	1578	1438.6

a: AIS, adenocarcinoma in situ; MIA, minimally invasive adenocarcinoma; IA, invasive adenocarcinoma.
